# Bone-targeting AAV-mediated silencing of Schnurri-3 prevents bone loss in osteoporosis

**DOI:** 10.1038/s41467-019-10809-6

**Published:** 2019-07-04

**Authors:** Yeon-Suk Yang, Jun Xie, Dan Wang, Jung-Min Kim, Phillip W. L. Tai, Ellen Gravallese, Guangping Gao, Jae-Hyuck Shim

**Affiliations:** 1 Department of Medicine/Division of Rheumatology, University of Massachusetts Medical School, Worcester, MA 01605 USA; 2 Horae Gene Therapy Center, University of Massachusetts Medical School, Worcester, MA 01605 USA; 3 Department of Microbiology and Physiological Systems, University of Massachusetts Medical School, Worcester, MA 01605 USA; 4 Viral Vector Core, University of Massachusetts Medical School, Worcester, MA 01605 USA; 5 Li Weibo Institute for Rare Diseases Research, University of Massachusetts Medical School, Worcester, MA 01605 USA

**Keywords:** siRNAs, Gene therapy, Metabolic bone disease

## Abstract

RNAi-based bone anabolic gene therapy has demonstrated initial success, but many practical challenges are still unmet. Here, we demonstrate that a recombinant adeno-associated virus 9 (rAAV9) is highly effective for transducing osteoblast lineage cells in the bone. The adaptor protein Schnurri-3 (SHN3*)* is a promising therapeutic target for osteoporosis, as deletion of *shn3* prevents bone loss in osteoporotic mice and short-term inhibition of *shn3* in adult mice increases bone mass. Accordingly, systemic and direct joint administration of an rAAV9 vector carrying an artificial-microRNA that targets *shn3* (rAAV9-*amiR-shn3*) in mice markedly enhanced bone formation via augmented osteoblast activity. Additionally, systemic delivery of rAAV9-*amiR-shn3* in osteoporotic mice counteracted bone loss and enhanced bone mechanical properties. Finally, we rationally designed a capsid that exhibits improved specificity to bone by grafting the bone-targeting peptide motif (AspSerSer)_6_ onto the AAV9-VP2 capsid protein. Collectively, our results identify a bone-targeting rAAV-mediated gene therapy for osteoporosis.

## Abstract (web summary)

The adaptor protein SHN3 suppresses new bone formation by controlling osteoblast activity. Here, the authors show that ablation of SHN3 function, either genetically or by delivering an artificial miRNA via AAV9, rescues bone loss in osteoporotic mice, and show that engineering of the AAV9 capsid improves targeting to bone

## Introduction

Adult bone mass is determined by the balance between bone formation by osteoblasts and bone resorption by osteoclasts. Disturbances in this equilibrium to favor osteoclast-mediated resorption result in low bone density and deterioration of bone structure, which increase the risk of fractures^1^. Current leading osteoporosis therapies target osteoclasts to inhibit bone resorption, but these therapeutic agents are accompanied by numerous side effects, including atypical femoral fractures and osteonecrosis of the jaw^2^. Only two anabolic agents, parathyroid hormone^3^ and parathyroid hormone-related protein^4,5^, exist for promoting osteoblast function to treat patients with osteoporosis. However, these agents are costly, require frequent injections, and have the potential for promoting the development of osteosarcomas. Recently developed agents including the antisclerostin antibody^6^ and a small molecule inhibitor of Cathepsin K^7^ can increase bone mass and reduce fracture risk in osteoporosis. However, these drugs show adverse cardiovascular events in clinical trials^8^. Thus, therapeutic agents that can treat osteoporosis without adverse effects are still an unmet need.

We previously identified the adaptor protein Schnurri-3 (SHN3) as a potent endogenous inhibitor of bone formation^9,10^. Mice lacking *shn3* in osteoblasts display a significant increase in bone mass. The SHN3 zinc-finger protein is encoded by the *Hivep3* gene in humans and mice, and is herein referred to as *shn3*. It controls osteoblast differentiation by promoting RUNX2 degradation^9^ and suppressing the Wnt signaling pathway by inhibiting ERK MAPK activity^11^. In addition, SHN3 inhibits the production of the proangiogenic factor SLIT3, which supports the CD31^high^endomucin^high^ vascular endothelium during bone formation^10^. Lastly, SHN3 deficiency is not associated with any observable phenotypes in nonskeletal tissues^10,12^. These properties together make SHN3 inhibition an attractive approach to promote bone formation to treat osteoporosis.

Adeno-associated virus (AAV) is a small (26 nm) nonenveloped parvovirus with a single-stranded genome of ~4.7 kb in length^13^. High transduction efficiency, persistent transgene expression, and lack of post-infection immunogenicity and pathogenicity make AAV an attractive viral vector for use in gene therapy^14^. The AAV genome encodes regulatory proteins (*Rep*) and structural capsid proteins (*Cap*) and is flanked by two inverted terminal repeats. Replacement of the *Rep* and *Cap* genes with a transgene of interest produces a replication-defective recombinant AAV (rAAV) genome that can transduce target tissues as a potent vector^15,16^. Second-generation self-complementary AAVs (scAAVs) that package a double-stranded DNA genome were engineered to bypass the rate-limiting second-strand synthesis step required for rAAV transgene expression^17–20^. As a result, scAAV vectors have enhanced transduction efficacies in vitro and in vivo. To date, AAV vectors have been evaluated in over 130 clinical trials worldwide^21^. However, AAV-based gene therapies for bone and joint disorders are limited^22^.

In this study, we demonstrate that among 14 conventional serotypes tested^23^, AAV9 is the most effective for in vitro and in vivo transduction of osteoblast lineage cells. Similar to bone accrual by tamoxifen-induced deletion of *shn3* in osteoblasts, systemic delivery of an rAAV9 packaged with a *Cre* recombinase transgene in mice harboring a floxed *shn3* allele can efficiently mediate *shn3* deletion in osteoblasts to increase bone accrual. Importantly, we provide a proof-of-concept demonstration that an rAAV vector that carries an artificial microRNA (*amiR*) targeting *shn3* can reverse bone loss and enhance bone quality in a mouse model of osteoporosis. Finally, to improve osteo-specific transduction, the bone-targeting peptide motif (AspSerSer)_6_^24^ was grafted onto the AAV9-VP2 capsid protein, resulting in significant reduction of transgene expression in nonbone peripheral organs.

## Results

### Transduction of AAV serotypes in bone and cartilage

**Fig. 1:**
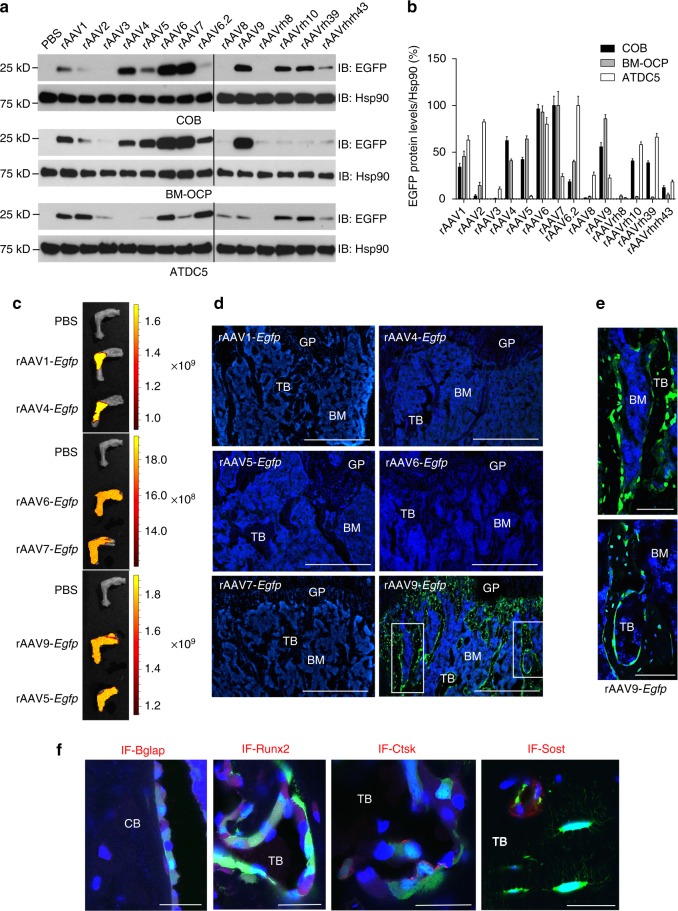
Identification of rAAV vectors that transduce bone cells in vitro and in vivo. **a**, **b** Calvarial osteoblasts (COB), bone marrow-derived osteoclast precursors (BM-OCP), or chondrogenic cells (ATDC5), were treated with PBS or 14 different AAV capsids packaged with the same *CB-Egfp* transgene. After 2 days, EGFP expression was assessed by immunoblotting with an anti-GFP antibody (**a**). The anti-Hsp90 antibody was used as a loading control. **b** Immunoblot quantification of EGFP protein was measured as a percentage of endogenous Hsp90 protein level by ImageJ software. **c**–**f** A single dose of 1 × 10^11^ genome copies of rAAV was intraarticularly (i.a.) injected into the knee joints of 2-month-old male mice, and EGFP expression in the hindlimb was monitored by IVIS-100 optical imaging 2 weeks post injection (**c**). Femurs were cryosectioned and EGFP expression was assessed by fluorescence microscopy (**d**). **e** High-magnification images of EGFP-expressing cells in the femur. Cryo-sectioned femurs were also immunostained for Bglap, Runx2, Ctsk, and Sost to identify osteoblasts, mature osteoclasts, and osteocytes, respectively (**f**). TB, trabecular bone; BM, bone marrow; GP, growth plate; CB, cortical bone. Scale bars: **d** 500 μm; **e** 100 μm; **f** 25 μm

We first aimed to identify the best AAV serotype for transducing cells of the bone and cartilage lineage in vitro. An scAAV vector construct expressing the enhanced green fluorescent protein (*Egfp*) reporter gene was packaged into 14 conventional AAV capsids (AAV1, AAV2, AAV3, AAV4, AAV5, AAV6, AAV6.2, AAV7, AAV8, AAV9, AAVrh.8, AAVrh.10, AAVrh.39, and AAVrh.43)^25^ and incubated with mouse calvarial osteoblasts (COBs), bone marrow-derived osteoclast precursors (BM-OCPs), and chondrocyte progenitor cells (ATDC5s) at three different MOIs. Expression of EGFP in transduced cells was assessed by immunoblotting with an anti-EGFP antibody (Fig. 1a, b) and fluorescence microscopy (Supplementary Fig. 1). Eight AAV serotype vectors, rAAV1, rAAV4, rAAV5, rAAV6, rAAV7, rAAV9, rAAVrh10, and rAAVrh39 were able to transduce COBs (Fig. 1a). Among these, rAAV1, rAAV4, rAAV5, rAAV6, rAAV7, and rAAV9 also transduced BM-OCPs, while rAAV1, rAAV6, rAAVrh.10, and rAAVrh.39 transduced ATDC5 cells. Interestingly, rAAV2 and rAAV6.2 transduced ATDC5 cells efficiently, but poorly in COBs.

We note that the transduction efficiencies of rAAVs in vitro are scarcely predictive of their in vivo performance. This phenomenon is due to the presence of multiple physiological barriers, including the route of administration, serum factors, circulating neutralizing antibodies, and extracellular barriers^25^. Therefore, in vivo evaluation to assess the ability of serotypes to transduce-specific tissues or cell types is required. To examine the tropism of AAV capsids to articular cartilage and bone, eight rAAV-*Egfp* vectors selected from the in vitro screen (rAAV1, rAAV4, rAAV5, rAAV6, rAAV7, rAAV9, rAAVrh.10, and rAAVrh.39) were intraarticularly (i.a.) injected into knee joints of 2-month-old mice, and EGFP expression was monitored by IVIS-100 optical imaging of whole bone (Fig. 1c) and by fluorescence microscopy of bone cryosections (Fig. 1d–f and Supplementary Fig. 2). Surprisingly, there was little to no expression of EGFP in the growth plate (Fig. 1d) or the articular cartilage (Supplementary Fig. 2a) for the majority of capsids. This discrepancy may be due to the poor accessibility of rAAV vectors to chondrocytes, which are embedded in the avascular microenvironment of these structures. Alternatively, vectors may simply exhibit a lower infectivity of primary chondrocytes in adult mice. While most rAAV vectors showed relatively higher EGFP expression in the adjacent muscle (Supplementary Fig. 2b), EGFP expression in trabecular and cortical bone was only detected in rAAV9-treated hindlimbs (Fig. 1d, e and Supplementary Fig. 2b). Specifically, rAAV9-mediated EGFP expression in the femur was detected in osteoblasts and osteoclasts on the bone surface, and in osteocytes embedded in the bone matrix (Supplementary Fig. 2c). Notably, EGFP expression was also detected in a subset of Osteocalcin- or Runx2-positive osteoblasts, Cathepsin K-positive osteoclasts, and Sclerostin-positive osteocytes, demonstrating the ability of AAV9 to transduce osteoblasts, osteoclasts, and osteocytes in vivo (Fig. 1f).

### Systemic delivery of rAAV9 can target osteoblasts

**Fig. 2:**
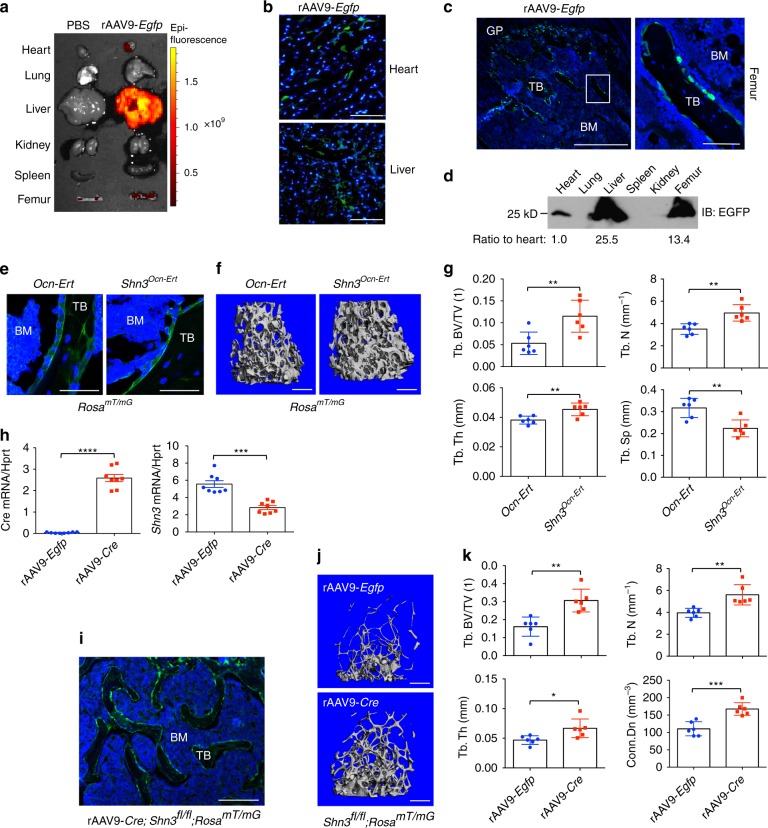
Inducible deletion of *shn3* in osteoblasts increases bone accrual in adult mice. **a**–**d** A single dose of 4 × 10^11^ genome copies of rAAV9-*Egfp* was intravenously (i.v.) injected into 2-month-old male mice and EGFP expression in individual tissues was monitored by IVIS-100 optical imaging 2 weeks post injection. *y*-axis, radiant efficiency (p/s/cm^2^/sr/μW/cm^2^) (**a**). EGFP expression was assessed by fluorescence microscopy on cryo-sectioned heart, liver (**b**), and femur (**c**). A high-magnification image of EGFP-expressing osteoblasts in the femur (**c**, right). Immunoblot of tissue lysates with an anti-GFP antibody (**d**). **e**–**g** Two-month-old female *Ocn-Ert;Rosa*^*mT/mG*^ and *Shn3*^*Ocn-Ert*^*;Rosa*^*mT/mG*^ mice were treated with 100 mg/kg of tamoxifen for 5 consecutive days; 2 months later, femurs were cryo sectioned to identify EGFP-expressing osteoblasts (**e**). Femoral trabecular bone mass was assessed by microCT. Representative 3D reconstruction (**f**) and relative quantification (**g**) are displayed. Trabecular bone volume/total volume (Tb. BV/TV), trabecular thickness (Tb.Th), trabecular number per cubic millimeter (Tb.N), and trabecular space (Tb. Sp) (*n* = 6 per group). **h**–**k** A single dose of 4 × 10^11^ genome copies of rAAV9-*Egfp* or rAAV9-*Cre* was i.v. injected into 3-month-old male *Shn3*^*fl/fl*^*;Rosa*^*mTmG*^ mice. After 2 months, *cre* and *shn3* mRNA levels were assessed in tibial bone RNA and normalized to *hprt* (**h**). Fluorescence microscopy was performed on cryo-sectioned femurs to identify EGFP-expressing cells (**i**) and femoral trabecular bone mass was assessed by microCT. Representative 3D reconstruction (**j**) and relative quantification (**k**) are displayed. Trabecular bone volume/total volume (Tb. BV/TV), trabecular thickness (Tb. Th), trabecular number per cubic millimeter (Tb. N), and connective density (Conn. Dn) (*n* = 6 per group). Scale bars: **b** 50 μm; **c** 500 μm (left), 75 μm (right); **e** 25 μm; **f**, **j** 1 mm; **i** 250 μm. Values represent mean ± SD: *P < 0.05; **P < 0.01; and ***P < 0.001 by an unpaired two-tailed Student’s *t*-test (**g**, **h**, **k**)

To test AAV9’s ability to target bone tissue in vivo when delivered systemically, rAAV9-*Egfp* was injected into 2-month-old mice and the tissue distribution of rAAV9 was assessed by EGFP expression 2 weeks post injection. Whole body and individual organ imaging of treated mice showed that EGFP expression was the highest in the liver and hindlimbs (Fig. 2a and Supplementary Fig. 3a, b). Expression in the heart and femur was modest, while expression in the lung, kidney, and spleen was not detected. Expression of EGFP in the heart, liver, femur, and vertebrae was further confirmed by fluorescence microscopy (Fig. 2b, c and Supplementary Fig. 3c, d) and immunoblot analysis (Fig. 2d). As observed in i.a. injected hindlimbs, EGFP protein was primarily expressed in endosteal osteoblasts and osteocytes in cortical and trabecular bones, but not in the ligament, articular cartilage, growth plate, periosteal osteoblasts, and patella (Fig. 2c and Supplementary Fig. 4). These results demonstrate that systemically delivered rAAV9 vector targets osteoblast lineage cells residing in the endosteal bone.

### *shn3* deletion promotes bone formation in adult mice

Our previous studies identified SHN3 as a potent suppressor of osteoblast differentiation^9,11,12^. Specifically, conditional deletion of a floxed *shn3* allele with *Cre*-deleter mice targeting mesenchymal stem cells (*Shn3*^*Prx1*^)^12^ or mature osteoblasts/osteocytes (*Shn3*^*Dmp1*^)^10^ results in a significant increase in bone accrual. To examine the effects of short-term inhibition of SHN3 on bone formation, we generated an inducible, osteoblast-specific *shn3*-knockout mice by crossing *Shn3*^*fl/fl*^ mice with osteocalcin-*CreERT* mice expressing a tamoxifen-induced Cre recombinase in mature osteoblasts^26^ (*Shn3*^*Ocn-Ert*^). These mice were further crossed with the *Cre*-reporter *Rosa*^*mT/mG*^ mice to visualize Cre-expressing cells^27^ (*Shn3*^*Ocn-Ert*^; *Rosa*^*mT/mG*^). Treatment of *Shn3*^*Ocn-Ert*^; *Rosa*^*mT/mG*^ mice with tamoxifen resulted in the expression of GFP in mature osteoblasts on the surface of trabecular and cortical bones, indicating osteoblast-specific deletion of *shn3* (Fig. 2e). Accordingly, these mice showed a significant increase in trabecular bone mass and a mild increase in cortical bone thickness compared to tamoxifen-treated control mice (Fig. 2f, g and Supplementary Fig. 5). These results demonstrate that inducible deletion of *Shn3* in mature osteoblasts is sufficient to increase bone mass in adult mice.

To provide proof-of-concept demonstration that systemically delivered rAAV9 can direct the deletion of *snh3* in osteoblast lineage cells to promote bone formation, we generated a Cre-encoding rAAV9 vector (rAAV9-*Cre*, Supplementary Fig. 6a) to serve as the facilitator for Cre-recombination in *Shn3*^*fl/fl*^ mice. We first treated rAAV9-*Cre* in cultured COBs isolated from *Shn3*^*fl/fl*^ mice (Supplementary Fig. 6b), and as expected, rAAV9-mediated Cre expression in *Shn3*^*fl/fl*^ COBs resulted in the deletion of *shn3* and enhanced osteoblast differentiation (Supplementary Fig. 6c–e). We next injected rAAV9-*Cre* into 2-month-old *Shn3*^*fl*/fl^;*Rosa*^*mT/mG*^ mice via i.v. administration. Two months after injection, expression of *Cre* mRNA in the femur (Fig. 2h, left) and Cre-mediated expression of EGFP protein in osteoblast lineage cells residing on the bone surface (Fig. 2i) were validated by RT-PCR and fluorescence microscopy, respectively. Compared to rAAV9-*Egfp-*treated femurs, rAAV9-*Cre*-treated femurs showed a significant decrease in *shn3* mRNA levels (Fig. 2h, right) and an increase in relative trabecular bone mass in the femur and lumbar vertebrae (Fig. 2j, k and Supplementary Fig. 7a). However, cortical bone at the femoral diaphysis showed no significant change in thickness (Supplementary Fig. 7b). These results demonstrate that systemically delivered rAAV9-*Cre* in *Shn3*^*fl/fl*^ mice targets osteoblast lineage cells and mediates *shn3* deletion to increase bone mass. Importantly, our results demonstrate that rAAV9-mediated transgene delivery to osteoblast lineage cells can dramatically alter bone physiology.

### rAAV9-mediated silencing of *shn3* promotes bone formation

**Fig. 3:**
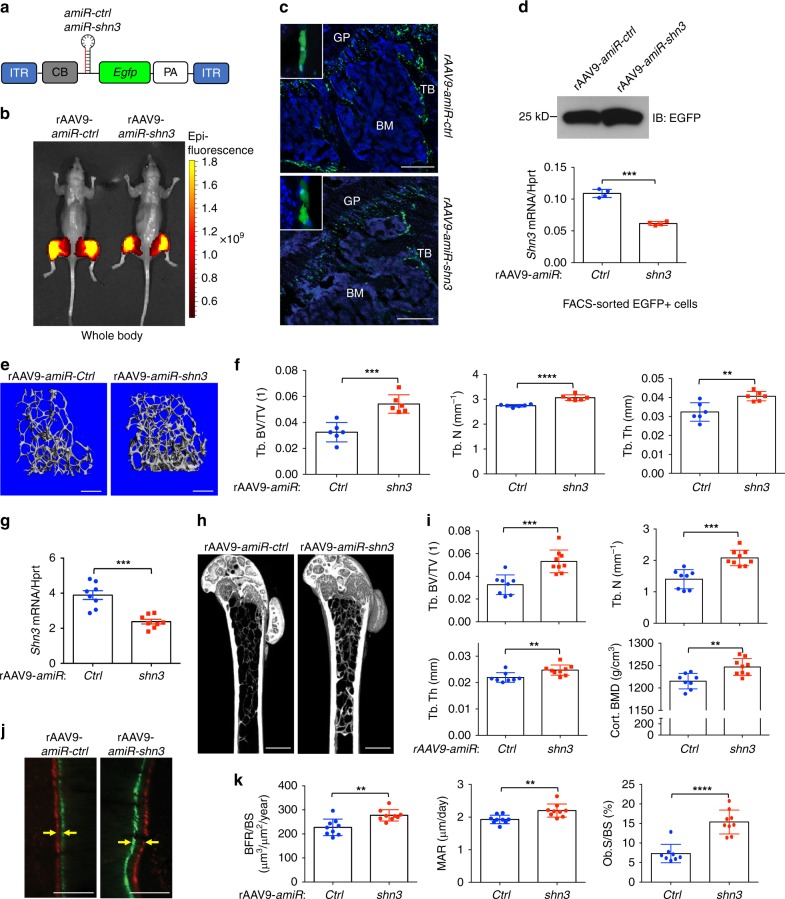
Silencing of SHN3 by systemically delivered rAAV9 promotes bone formation. **a** Diagram of the rAAV9 construct containing a CMV enhancer/chicken β-actin promoter (*CB*), *amiR-ctrl* or *amiR-shn3*, *an Egfp* reporter gene (*EGFP*), *β-globin* polyA sequence (PA), and inverted terminal repeat (ITR). **b**–**d** Two weeks after i.a. injection of rAAV9 carrying *amiR-ctrl* or *amiR-shn3* into knee joints of 2-month-old male mice, EGFP expression was assessed by IVIS-100 optical imaging (**b**) and fluorescence microscopy of cryo-sectioned femurs (**c**). Levels of EGFP protein (**d**, top) and *shn3* mRNAs normalized to *hprt* (**d**, bottom) were assessed in FACS-sorted EGFP-expressing cells from the femur. **e**, **f** Two months after i.a. injection of rAAV9 carrying *amiR-ctrl* or *amiR-shn3* into knee joints of 2-month-old female mice, femoral trabecular bone mass was assessed by microCT. Representative 3D reconstruction (**e**) and relative quantification (**f**) are displayed (*n* = 6 per group). **g**–**k** A single dose of 4 × 10^11^ genome copies of rAAV9 carrying *amiR-ctrl* or *amiR-shn3* were i.v. injected into 3-month-old female mice. After 2 months, *shn3* mRNA levels were assessed in the tibial bone RNA and normalized to *hprt* (**g**, *n* = 8 per group). Femoral trabecular bone mass was assessed by microCT. Representative 3D reconstruction (**h**) and relative quantification (**i**) are displayed (*n* = 8 per group). Representative calcein/alizarin red labeling (**j**) and relative histomorphometric quantification of BFR/BS, MAR, and Ob.S/BS are displayed (**k**). Arrows indicate the distance between calcein and alizarin red labeling. BFR/BS, bone formation rate/bone surface; MAR, mineral apposition rate; Ob.S/BS, osteoblast surface/bone surface. GP, growth plate; BM, bone marrow; TB, trabecular bone. Scale bars: **c** 250 μm; **e**, **h** 1 mm; **j** 50 μm. Values represent mean ± SD: **P < 0.01; ***P < 0.001; and ****P < 0.0001 by an unpaired two-tailed Student’s *t*-test (**f**, **g**, **i**, **k**)

High levels of AAV-delivered shRNAs can induce cytotoxicity by perturbing the RNAi machinery or exhibit significant off-target silencing^28,29^. Embedding the guide strand of a small silencing RNA into the mouse miR-33-derived miRNA scaffold limits shRNA-related toxicity and enables efficient gene knockdown, while reducing off-target silencing by tenfold compared to conventional shRNA constructs^30^. We therefore engineered amiR cassettes to target *shn3* (*amiR-shn3*) or a control (*amiR-ctrl*). In this design, the amiR is inserted intronically between the *CB* promoter and the *Egfp* reporter gene (Fig. 3a), which allows for visual tracking of positively transduced cells or tissues. First, we validated gene knockdown efficacy of the amiR cassettes by transfecting Myc-tagged mouse *shn3* into HEK293 cells along with plasmids encoding *amiR-shn3 or amiR-ctrl* plasmids (Supplementary Fig. 8a). Constructs were then packaged into AAV9 capsids. Treatment with rAAV9-*amiR-shn3* or rAAV9-*amiR-ctrl* resulted in positively transduce COBs in vitro (Supplementary Fig. 8b). Compared to *amiR-ctrl*-treated COBs, treatment with rAAV9-*amiR-shn3* resulted in ~50% reduction of *shn3* mRNA levels, and relative increases in *ibsp* expression and mineralization (Supplementary Fig. 8c, d).

To examine the ability of rAAV9-*amiR-shn3* to enhance bone-anabolic activity in vivo, vector was injected via i.a. administration into knee joints of 2-month-old mice. Two months following treatment, EGFP expression in hindlimbs and femurs was examined by IVIS optical imaging (Fig. 3b) and fluorescence microscopy (Fig. 3c), respectively. Importantly, FACS-sorted EGFP-expressing cells isolated from the femur showed ~50% reduction of *shn3* mRNA levels (Fig. 3d). Compared to *amiR*-*ctrl*-treated femurs, *amiR*-*shn3*-treated femurs showed a significant increase in relative trabecular bone mass (Fig. 3e, f). These results demonstrate that local delivery of rAAV9-*amiR-shn3* is effective in knocking down SHN3 expression in osteoblast lineage cells, and in turn, increases bone mass in vivo.

We next tested whether rAAV9-*amiR-shn3* could promote in vivo bone-anabolic activity following systemic delivery. Two months after i.v. administration into 3-month-old mice, EGFP expression was predominantly detected in the hindlimbs, liver, and heart as expected (Supplementary Fig. 9a–c). Femurs transduced by rAAV9-*amiR-shn3* displayed ~50% reduction of *shn3* mRNA levels (Fig. 3g) and a significant increase in trabecular bone mass of the femur and lumbar vertebrae (Fig. 3h, i and Supplementary Fig. 9d). No significant change in cortical bone thickness at the femoral diaphysis between treatment groups was observed (Supplementary Fig. 9e). Likewise, in vivo osteoblast activity was increased in the trabecular bone in the metaphysis of these mice, as shown by the greater bone formation rate (BFR), mineral apposition rate (MAR), and osteoblast surface per bone surface (Ob.S/BS) of rAAV9-*amiR-shn3*-treated mice (Fig. 3j, k). However, the number of tartrate-resistant acid phosphatase (TRAP)-positive osteoclasts and serum levels of the bone resorption marker C-terminal telopeptide type I collagen (CTX) were unchanged in these mice (Supplementary Fig. 9f, g). These results demonstrate that systemic delivery of rAAV9-*amiR-shn3* reduced *shn3* expression in osteoblast lineage cells, augmented osteoblast activity, and increased bone mass without any alteration in osteoclast number and function in vivo. Thus, the rAAV9-*amiR-shn3* vector may be useful for the treatment of osteoporosis as a potent bone anabolic agent.

### Effects of rAAV9-mediated silencing of *Shn3* in osteoporosis

**Fig. 4:**
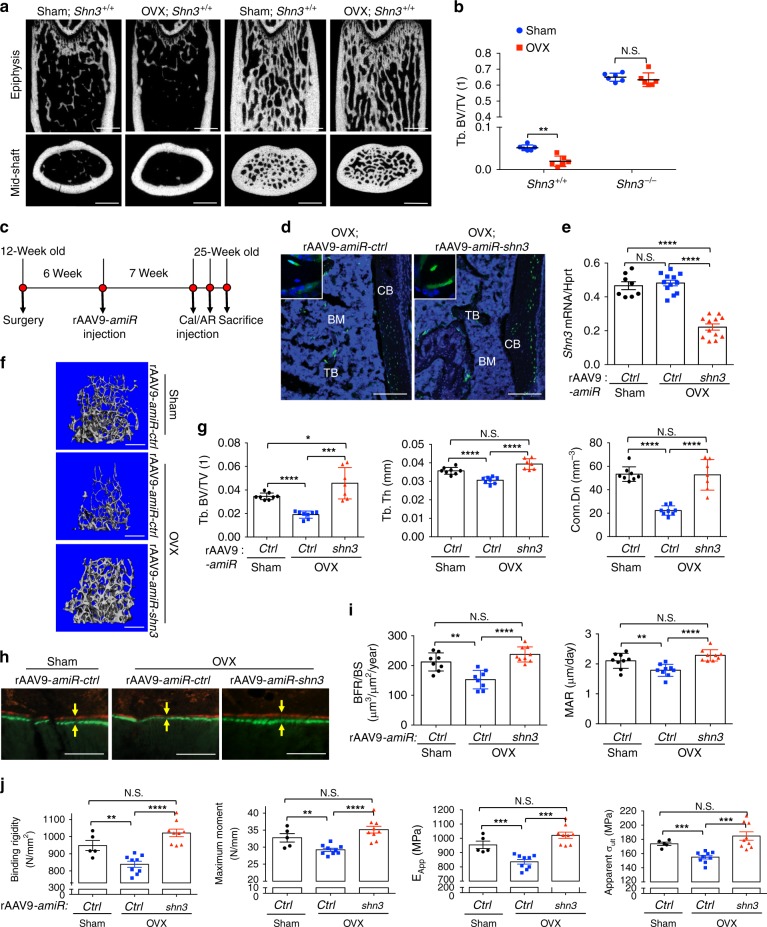
rAAV9-mediated silencing of *shn3* prevents bone loss in a mouse model of osteoporosis. **a**, **b** Sham or OVX surgery was performed on 3-month-old female *Shn3*^*+/+*^ *and Shn3*^*−/−*^ mice and 2 months later, femoral trabecular bone mass was assessed by microCT. Representative images of the femur (**a**) and relative BV/TV (**b**) are displayed (*n* = 6 per group). **c**–**j** Diagram of the study and treatment methods (**c**). Sham or OVX surgery was performed on 3-month-old female mice and 6 weeks later, a single dose of 4 × 10^11^ genome copies of rAAV9 carrying *amiR-ctrl* or *amiR-shn3* was i.v. injected. Seven weeks after injection, femurs were cryo sectioned to identify EGFP-expressing cells (**d**). *shn3* mRNA levels in tibial bone are displayed after normalization to *hprt* (*n* = 8–12 per group) (**e**). Femoral trabecular bone mass was assessed by microCT. Representative 3D reconstruction (**f**) and relative quantification (**g**) are displayed (*n* = 7–8 per group). Representative images of calcein/alizarin red labeling (**h**) and relative histomorphometric quantification of BFR/BS and MAR (**i**). Arrows indicate the distance between calcein and alizarin red labeling. Femoral biomechanical properties, including bending rigidity, bending moment, apparent bending modulus, and apparent bending stress were quantified (*n* = 5–9 per group) (**j**). Scale bars: **a**, **f** 1 mm; **d** 250 μm; and **h** 50 μm. Values represent mean ± SD: N.S., not significant; *P < 0.05; **P < 0.01; ***P < 0.001; and ****P < 0.0001 by an unpaired two-tailed Student’s *t*-test (**b**) and one-way ANOVA test (**e**, **g**, **i**, **j**)

Inhibition of WNT antagonists has been recognized as a new approach for therapeutic intervention in patients with osteoporosis^6^. Our previous studies identified SHN3 as an inhibitor of osteoblast differentiation via perturbation of Wnt signaling^9,11,12^. Ovariectomized (OVX) mice serve as established models for estrogen deficiency-induced osteoporosis^31^. To further establish that inhibition of SHN3 may be an attractive target to promote bone formation as a therapy for osteoporosis, 3-month-old female mice lacking SHN3 (*Shn3*^*−/−*^) were subjected to ovariectomies, and bone mass was assessed by microCT 2 months post surgery. While OVX surgery induced a significant reduction in trabecular bone mass in WT mice, OVX-induced bone loss was completely prevented by *shn3* deletion, as trabecular bone mass was comparable between sham-*Shn3*^*−/−*^ mice and OVX-*Shn3*^*−/−*^ mice (Fig. 4a, b). Thus, targeting *shn3* may have therapeutic potential to prevent bone loss in osteoporosis.

To test the therapeutic effects of rAAV9-*amiR-shn3* in osteoporosis, sham or OVX surgery was conducted on 3-month-old wild-type female mice, and vector was i.v. injected 6 weeks post surgery (Fig. 4c). After 7 weeks, treated femurs showed efficient transduction, leading to ~50% knockdown of *shn3* (Fig. 4d, e). As expected, *amiR-ctrl*-expressing OVX mice showed a significant decrease in trabecular bone mass in the femur and lumbar vertebrae relative to sham mice (Fig. 4f, g and Supplementary Fig. 10a, b). However, when treated with rAAV9-*amiR-shn3*, bone loss was completely reversed in the femur and lumbar vertebrae of OVX mice, as shown by greater trabecular BV/TV, thickness, and connectivity density. No significant change in cortical bone thickness at the diaphysis between all three groups was observed (Supplementary Fig. 10c). Likewise, femoral BFR and MAR were increased in these mice relative to *amiR-ctrl*-expressing OVX mice, demonstrating enhanced osteoblast activity in vivo (Fig. 4h, i). Notably, *shn3* silencing by rAAV9*-amiR-shn3* does not alter osteoclast function in vivo as the number of TRAP-positive osteoclasts and bone resorption activity are comparable between OVX mice expressing *amiR-ctrl* and *amiR-shn3* (Supplementary Fig. 10d, e). Finally, biomechanical testing showed that the strength and stiffness of femurs were considerably protected from OVX-induced bone loss of mice treated with rAAV9-*amiR-shn3* (Fig. 4j), suggesting that rAAV9-mediated silencing of *shn3* improves clinically meaningful endpoints in osteoporotic mice. Taken together, these results demonstrate that systemically delivered rAAV9-*amiR-shn3* promotes bone formation and enhances clinically relevant mechanical properties of bone after the onset of OVX-induced osteoporosis.

### Generation of a novel bone-tropic capsid of rAAV9

**Fig. 5:**
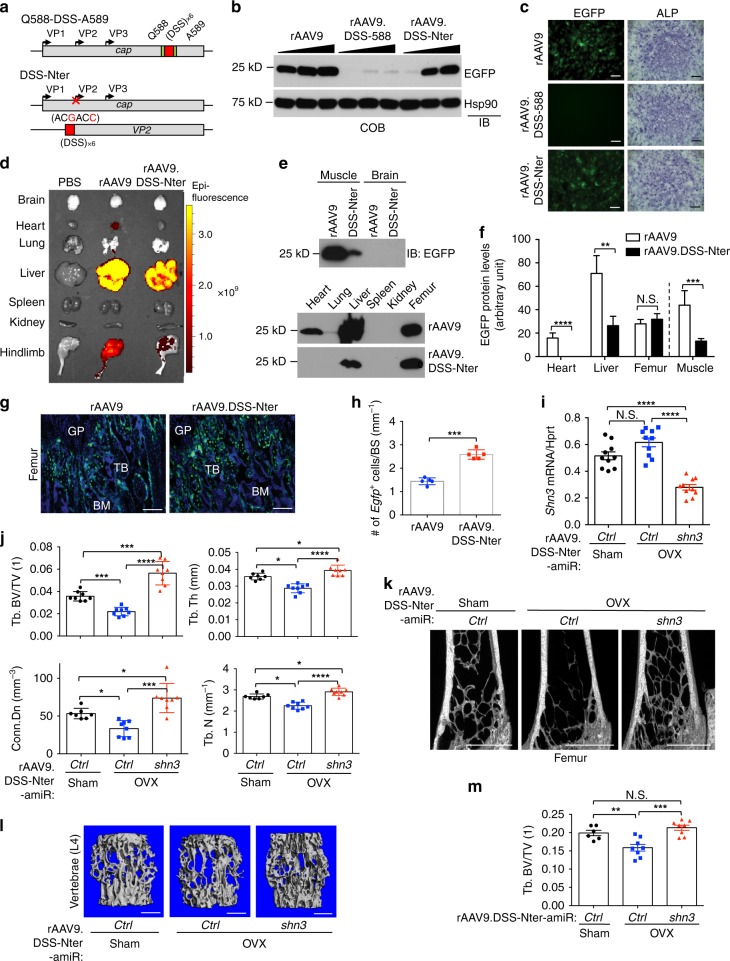
Effect of rAAV9.DSS-Nter-*amiR*-*shn3* in a mouse model of osteoporosis. **a** Diagram of constructs for rationally designed bone-specific AAV capsids. The bone-targeting peptide motif (DSS, red) was inserted into the AAV9 capsid between Q588 and A589 (DSS-588) or at the N-terminus of AAV9-VP2 (DSS-Nter). cap: capsid proteins. **b**, **c** Two days after infection with different concentrations of rAAV9, rAAV9.DSS-588, or rAAV9.DSS-Nter, COBs were cultured under osteogenic conditions for 6 days. EGFP expression was assessed by immunoblotting with an anti-GFP antibody (**b**) or fluorescence microscopy (**c**, left). ALP activity was assessed by fast blue staining (**c**, right). **d**–**h** A single dose of 4 × 10^11^ genome copies of rAAV9 or rAAV9.DSS-Nter was i.v. injected into 2-month-old male mice. EGFP expression in individual tissues was monitored by IVIS-100 optical imaging 2 weeks post injection (**d**). Immunoblotting shows EGFP expression in tissue lysates (**e**) and relative quantification (**f**, *n* = 3 per group). Fluorescence microscopy was performed on cryo-sectioned femurs to identify EGFP-expressing cells (**g**) and the number of EGFP-expressing cells per bone surface in femurs were quantified (**h**, *n* = 5 per group). **i**–**m** Sham or OVX surgery was performed on 3-month-old female mice. After 6 weeks, a single dose of 4 × 10^11^ genome copies of rAAV9.DSS-Nter carrying *amiR-ctrl* or *amiR-shn3* was i.v. injected. Seven weeks after injection, *shn3* mRNA levels were assessed in the tibial bone (**i**, *n* = 10 per group). Trabecular bone mass in the femur and lumbar vertebrae was assessed by microCT. Quantification (**j**, **m**) and representative 3D reconstruction (**k**, **l**) are displayed (*n* = 6 ~ 8 per group). GP, growth plate; BM, bone marrow; TB, trabecular bone. Scale bars: **g** 100 μm; **c**, **k**, **i** 1 mm. Values represent mean ± SD: N.S., not significant; *P < 0.05; **P < 0.01; ***P < 0.001; and ****P < 0.0001 by an unpaired two-tailed Student’s *t*-test (**f**, **h**) and one-way ANOVA test (**i**, **j**, **m**)

As demonstrated above, the majority of rAAV9 targets nonbone peripheral tissues, limiting the specificity of the rAAV9-*amiR-shn3* if delivered systemically. Since newly developed agents for osteoporosis, such as antisclerostin antibody (Romosozumab) and the small molecule inhibitor of Cathepsin K (Odanacatib) show off-target cardiovascular and cerebrovascular events in clinical trials^8,32^, a bone-specific treatment strategy must be developed. Thus, we aimed to detarget transduction from nonrelevant tissues by modifying the AAV9 capsid through a rational design approach to avoid potential nonskeletal side effects of rAAV9-mediated gene therapy. A previous study reported that the bone-targeting peptide motif, ((AspSerSer)_6_, DSS) was effective in directing an osteogenic siRNA-encapsulated liposome to the bone-forming surface, where osteoblast lineage cells typically reside^24^. Thus, to improve the ability of rAAV9 to target osteoblast lineage cells, DSS-encoding DNA sequences were inserted into either of two capsid positions that are amendable to peptide grafting^33^, at the loop IV domain between glutamine 587 and alanine 588 (AAV9.DSS-588) or at the N-terminus of VP2 (AAV9.DSS-Nter) (Fig. 5a). To test whether DSS insertion affects production of rAAV9, genome titers (GCs) of vectors packaged into prototypical AAV9, AAV9.DSS-588, or AAV9.DSS-Nter capsids were assessed by droplet digital PCR (ddPCR), demonstrating ~50% reduction in AAV9.DSS-588 or AAV9.DSS-Nter GCs compared to wild-type AAV9 GCs (Supplementary Fig. 11a). Next, vectors were infected into COBs, and EGFP expression was assessed by immunoblotting (Fig. 5b) or fluorescence microscopy (Fig. 5c, left). Compared to rAAV9, the rAAV9.DSS-Nter showed a modest reduction of infectivity at lower MOI (10^9^ GC/mL), whereas little EGFP expression was detected in COBs treated with rAAV9.DSS-588 (Fig. 5b, c, left). Similarly, when treating mice via i.a. injections, rAAV9 and rAAV9.DSS-Nter strongly transduced femurs as before (Supplementary Fig. 11b). In contrast, the rAAV9.DSS-588 yielded little to no transgene expression. In addition, treating COBs and BM-OCPs with rAAV9 and rAAV9.DSS vectors did not significantly alter respective osteoblast or osteoclast differentiation markers (Fig. 5c, right and Supplementary Fig. 11c–g), suggesting that treatment with rAAV9s does not lead to any vector-related adverse effects.

Since the AAV9.DSS-Nter engineered capsid still retained full transduction activity in vitro, we next tested the capsid for bone-targeting activity in vivo. As before, rAAV9-*Egfp* or rAAV9.DSS-Nter-*Egfp* vectors were i.v. injected into 2-month-old mice and their tissue distributions were assessed by EGFP expression 2 weeks post injection. Mice treated with rAAV9.DSS-Nter yielded EGFP expression levels in nonbone tissues that were comparatively less than those treated with rAAV9 (almost no expression in the heart, ~55% reduction in the liver, and ~75% reduction in the hindlimb) (Fig. 5d and Supplementary Fig. 12a, b). These results were supported by immunoblot and fluorescence microscopy data, which showed a lack of detectable EGFP in the heart, and a significant reduction in the liver and muscle among the rAAV9.DSS-Nter injection groups (Fig. 5e, f and Supplementary Fig. 12c). Importantly, expression in femurs and lumbar vertebrae were relatively comparable between treatment groups (Fig. 5e–g and Supplementary Fig. 12d). Similar to rAAV9-treated femurs, EGFP expression in rAAV9.DSS-Nter-treated femurs was detected in a subset of Runx2-positive osteoblasts, Cathepsin K-positive osteoclasts, and Sclerostin-positive osteocytes (Supplementary Fig. 12e). Intriguingly, the number of EGFP-expressing cells on the bone surface in rAAV9.DSS-Nter-treated femurs was increased relative to those in rAAV9-treated femurs when EGFP-expressing bone marrow cells were excluded for quantification (Fig. 5h). These results demonstrate that the engineered VP2 capsid protein fused with the bone-targeting peptide motif (DSS) improves the bone tropism of rAAV9 in part by detargeting delivery to nonskeletal tissues.

We next examined the capacity for the AAV9.DSS-Nter bone-tropic capsid to deliver the therapeutic *amiR-shn3* transgene (rAAV9.DSS-Nter-*amiR-shn3*) to OVX mice. Sham or OVX surgery was conducted on 3-month-old female mice and rAAV9.DSS-Nter-*amiR-shn3* was i.v. injected 6 weeks post surgery. Seven weeks after injection, animals exhibited strong EGFP expression in the liver, hindlimbs, and tails (Supplementary Fig. 13a), and in osteoblast lineage cells of femurs (Supplementary Fig. 13b), indicating robust transduction. Levels of *shn3* mRNAs were markedly reduced in *amiR-shn3-*expressing OVX femurs relative to *amiR-ctrl-*expressing sham or OVX femurs (Fig. 5i). While *amiR-ctrl*-expressing OVX mice showed a significant reduction in trabecular bone mass of femurs and lumbar vertebrae relative to sham mice, bone loss among OVX mice treated with rAAV9.DSS-Nter-*amiR-shn3* was significantly reversed (Fig. 5j–m and Supplementary Fig. 13c). No significant change in cortical bone thickness at the diaphysis between all three groups was observed (Supplementary Fig. 13d). Taken together, these results demonstrate that delivery of *amiR-shn3* by the bone-tropic AAV9.DSS-Nter capsid can counteract bone loss in osteoporosis.

## Discussion

Due to their lack of pathogenicity, AAV vectors have a long track record for safety and efficacy in relevant preclinical and clinical studies^34^. In addition, AAV vectors are capable of persisting in post-mitotic cells of host tissues for years^35,36^. For instance, a nonhuman primate study demonstrated that animals treated with a single-dose, intramuscular administration of *an rAAV-Epo* vector exhibited transgene persistence after more than 6 years^37^. In contrast, nonviral vectors such as nanoparticles and liposomes can be rapidly degraded, cleared in the circulation, have short biological half-lives, and generally exhibit nonspecific uptake^38^. In this study, we have demonstrated that rAAV9 serotype can target osteoblast lineage cells to drive RNAi-mediated silencing of *shn3*, which we show has a clinical significance as a genetic target to prevent bone loss and osteoporosis. Importantly, we now describe a promising platform for rAAV-based gene therapy for bone. Nonetheless, the long-term therapeutic outcomes of rAAV-mediated gene silencing in treating osteoporosis requires further investigation.

rAAV9-mediated gene therapy is currently the leading platform for treating neurological disorders^39^, in part, due to rAAV9’s ability to traverse the blood–brain barrier to target the central nervous system (CNS) when administered i.v.^40^. In addition, rAAV9 has been shown to transduce various peripheral tissues systemically, such as liver, retina, and striated muscles in adult mice^41,42^. Despite previous studies showing that in utero and calvarial administration of rAAV9 can transduce bone tissue in fetal and neonatal mice^41,43^, its ability to transduce cells within the adult bone tissue has gone largely unexplored. To our knowledge, this study is the first demonstration that systemic delivery of rAAV9 to adult mice can transduce osteoblasts, osteocytes, and osteoclasts in bone, while articular cartilage, growth plate, and ligament, and bone marrow are relatively refractory to rAAV9 transduction.

SHN3 is a promising therapeutic target for osteoporosis, evident by our findings that, (1) deletion of *shn3* completely prevents bone loss in a mouse model for osteoporosis and (2) short-term inhibition of SHN3 in adult mice increases bone formation. In addition, our proof-of-concept study demonstrated that a systemically delivered rAAV9.*amiR-shn3* vector can promote bone formation to fully reverse bone loss and enhance bone mechanical properties in osteoporosis, suggesting that rAAV9-mediated silencing of *shn3* improves clinically meaningful endpoints. While RNAi-based strategies to promote bone formation as therapies have been described before, such drugs in the form of bone anabolic liposomes^24^ or lipid-based nanoparticles^44^ require repeated injections. We demonstrate that a single systemic injection of rAAV9-*amiR-shn3* is sufficient to increase bone accrual in mice.

Our study was partially guided by previous investigations that examined the therapeutic potential for anti-sclerostin antibodies^6^ to increase bone mass and to reduce fracture risk due to osteoporosis. Related clinical trials unfortunately showed off-target cardiovascular adverse effects^32^. Here, our data confirm that rAAV9 can extensively transduce nonbone tissues, including cardiac muscle at low levels (Fig. 2a, b). It was therefore apparent that potential cardiovascular adverse effects of rAAV9 in the heart and other nonbone organs be considered. In addition, the engineering of improved capsids and vectors for bone-specific gene therapy is fundamentally important, despite extensive clinical successes for rAAV9, and our results suggesting that systemic knockdown of *shn3* using rAAV9 only impacts bone formation. Our platform, which utilizes an amiR approach, has yet to be fully tested in relevant preclinical models. As such, we do not fully understand the extent of safety and efficacy in human patients. These concerns are especially critical considering that AAV9 can target a wide range of tissues and has the capacity to cross the blood–brain barrier to target the CNS. To address this downside to AAV9, we engineered a capsid by grafting the bone-targeting peptide motif onto AA9-VP2. This rationally designed capsid, named AAV9.DSS-Nter, exhibits remarkable reduced tropism to liver, heart, and muscle. Since the DSS peptide motif has a higher binding affinity to the osteoblast-enriched bone-formation surface than to the osteoclast-enriched bone-resorption surface^24^, display of the DSS peptide on the capsid protein may enhance rAAV9’s ability to transduce osteoblast lineage cells in vivo.

Systemic delivery of *amiR-shn3* or other therapeutic transgenes via rAAV9.DSS-Nter may have clinical utility for counteracting bone loss in other human diseases, such as inflammatory arthritis-induced bone loss. In addition, local delivery of therapeutic transgenes via rAAV9.DSS-Nter to areas of bone fracture may also be useful for enhancing bone healing. This possibility opens the door to future work that explores the therapeutic effects of rAAV-mediated gene therapy in the bone. We note that rAAV9-mediated silencing of *shn3* is more effective to increase trabecular bone mass at the metaphysis than cortical bone thickness at the femoral diaphysis. This outcome is likely due to relatively lower bone remodeling activity of cortical bone at the diaphysis. Future vector improvements to transduce exclusively osteoblast lineage cells such as using osteoblast-specific promoters in the AAV vector genome design or the further improvements to capsids will allow for even more precise bone-targeting rAAV vectors that can deliver therapeutic genes absent of any nonskeletal effects.

## Methods

### rAAV vector design and production

The artificial miRNA against mouse *shn3* gene was designed by using a custom Excel macro, which considers miR-33 scaffold design rules to generate optimized amiR cassettes. The tool will be shared upon request. Plasmids were constructed by Gibson assembly and standard molecular biology methods. DNA sequences for *amiR-33-ctrl* and *amiR-33-shn3* were synthesized as gBlocks and cloned into the intronic region of the pAAVsc-*CB6-Egfp* plasmid at the restriction enzyme sites (PstI and BglII)^45^. The pAAVsc-*CB6-Cre* was generated by replacing the *Egfp* reporter with Cre recombinase. Constructs were verified by sequencing. Our previous study showed that doxycycline-inducible expression of shRNA targeting mouse *shn3* in transgenic mice resulted in a decrease in *shn3* mRNA levels and a relative increase in bone mass^11^. The same targeting sequence was used to generate the *amiR-33-shn3* cassette. The pAAV-*amiR-ctrl* and pAAV-*amiR-shn3* constructs were packaged into AAV9 capsids. In addition, the pAAVsc-CB6-*Egfp* construct was packaged into AAV1 (1.8E + 13 GC/ml), AAV2 (1.5E + 12 GC/ml), AAV3 (6E + 12 GC/ml), AAV4 (6.5E + 12 GC/ml), AAV5 (2.4E + 13 GC/ml), AAV6 (8E + 12 GC/ml), AAV6.2 (8E + 12 GC/ml), AAV7 (1.5E + 13 GC/ml), AAV8 (7E + 12 GC/ml), AAV9 (1.5E + 13 GC/ml), AAVrh8 (8E + 12 GC/ml), AAVrh10 (8E + 12 GC/ml), AAVrh39 (1.0E + 13 GC/ml), and AAVrh43 (6E + 12 GC/ml) capsids. rAAV production was performed by transient transfection of HEK293 cells, purified by CsCl sedimentation, titered by ddPCR on a QX200 ddPCR system (Bio-Rad) using the *Egfp* prime/probe set as previously described^46^. The sequences of gBlocks and oligonucleotides for ddPCRare listed in Supplementary Table 1.

### Generation of bone-targeting rAAV9 vector

The DNA sequence encoding the bone-targeting peptide motif DSS (AspSerSer)_6_ was codon-optimized. To generate the Q588-DSS-A589 capsid (DSS-588), a plasmid expressing AAV2 *rep* gene and AAV9 *cap* gene (pAAV2/9) was modified to insert the DSS sequence into the AAV9 *cap* gene between the Q588 and A589 codons (pAAV2/9.Q588-DSS-A589). This plasmid was used in rAAV production (5–8E + 12 GC/ml). To generate the DSS-Nter capsid, we used a pair of plasmids. First, the start codon of VP2 in pAAV2/9 was mutated (ACG→ACC), so that only VP1 and VP3 are expressed (pAAV2/9.noVP2). In another plasmid, the DSS sequence was fused to the N-terminus of the AAV9-VP2 ORF. A Kozak sequence and ATG start codon were placed immediately upstream of the DSS sequence allowing for optimal expression driven by the CMV promoter [pcDNA.DSS-VP2(AAV9)]. The plasmids pAAV2/9.noVP2 and pcDNA.DSS-VP2(AAV9) were used in rAAV production (6–10E + 12 GC/ml).

### Cells

The chondrogenic ATDC5 cells were purchased from Sigma and cultured in DMEM/Ham’s F12 medium supplemented with 2% FBS, 2 mM l-glutamine and 1% penicillin/streptomycin. In addition, primary osteoprogenitors (COB) were isolated from calvaria of 5-day-old wild-type neonates (C57BL/6 J) using Collagenase type II (50 mg/ml, Worthington, LS004176) and Dispase II (100 mg/ml, Roche, 10165859001) and were maintained in α-MEM medium (Gibco) containing 10% FBS (Gibco), 2 mM l-glutamine (Corning), 1% penicillin/streptomycin (Corning), and 1% nonessential amino acids (Corning). COBs were differentiated with ascorbic acid (200 uM, Sigma, A8960) and β-glycerophosphate (10 mM, Sigma, G9422). Finally, bone marrow cells were flushed from the femurs and tibias of 2-month-old mice (C57BL/6 J), and cultured in petri dishes in α-MEM medium with 10% FBS and 20 ng/ml of M-CSF (R&D systems) to obtain bone BM-OCP. After 12 h, nonadherent cells were replated into tissue culture dishes and cultured in the same medium for 3 days. BM-OCPs then differentiated into osteoclasts in the presence of RAMKL (20 ng/ml; R&D systems) and M-CSF (20 ng/ml; R&D systems) for 6 days.

### Mice

*Shn3*^*−/−*^ mice^9^ *and Shn3*^*fl/fl*^ mice^47^ were generated as previously described and maintained on BALB/cJ and C57BL/6J background, respectively. Osteocalcin-ERT/Cre mice with tamoxifen-induced Cre recombinase expression in mature osteoblasts^48^ were crossed with *Shn3*^*fl/fl*^ mice to obtain *Shn3*^*fl/fl*^;*Ocn-ERT/Cre* mice (C57BL/6J). To label Cre-expressing cells, *Shn3*^*fl/fl*^;*Ocn-ERT/Cre* mice were further crossed with *Rosa*^*mT/mG*^ cre reporter mice (C57BL/6J)^27^. For postnatal activation of ERT/Cre, 100 mg/kg tamoxifen (Sigma) in corn oil (Sigma) was intraperitoneally injected to 2-month-old female mice once a day for 5 consecutive days.

Mouse genotypes were determined by PCR on tail genomic DNA. Primer sequences are available upon request. Control littermates were used and analyzed in all experiments. The animal protocols were reviewed and approved by the University of Massachusetts Medical School on animal care (IACUC), and were conformed to the NIH Guide for the Care and Use of Laboratory Animals.

### MicroCT analysis

MicroCT was used for qualitative and quantitative assessment of trabecular and cortical bone microarchitecture and performed by an investigator blinded to the genotypes of the animals under analysis. Femurs excised from the indicated mice were fixed with 10% neutral buffered formalin and scanned using a microCT 35 (Scanco Medical) with a spatial resolution of 7 μm. For trabecular bone analysis of the distal femur, an upper 2.1 mm region beginning 280 μm proximal to the growth plate was contoured. For cortical bone analysis of femur, a mid-shaft region of 0.6 mm in length was used. MicroCT scans of L4 spinal segments were performed using isotropic voxel sizes of 12 μm. Three-dimensional reconstruction images were obtained from contoured two-dimensional images by methods based on distance transformation of the binarized images. Alternatively, the Inveon multimodality 3D visualization program was used to generate fused 3D viewing of multiple static or dynamic volumes of microCT modalities (Siemens Medical Solutions USA, Inc). All images presented are representative of the respective genotypes (*n* > 5).

### Histology, histomorphometry, and immunofluorescence

For histological analysis, femurs and vertebrae were dissected from the mice treated with rAAVs vectors, fixed in 10% neutral buffered formalin for 2 days, and decalcified by 5% tetrasodium EDTA for 2–4 weeks. Tissues were dehydrated by passage through an ethanol series, cleared twice in xylene, embedded in paraffin, and sectioned at a thickness of 6 μm along the coronal plate from anterior to posterior. Decalcified femoral sections were stained with hematoxylin and eosin (H&E) or TRAP.

For dynamic histomorphometric analysis, 25 mg/kg calcein (Sigma, C0875) and 50 mg/kg alizarin-3-methyliminodiacetic acid (Sigma, A3882) dissolved in 2% sodium bicarbonate solution were subcutaneously injected into mice at 6 days interval. After fixed in 10% neutral buffered formalin for two days, undecalcified femur samples were embedded in methylmethacrylate and proximal metaphysis is sectioned longitudinally (5 μm) and stained with McNeal’s trichrome for osteoid assessment, toluidine blue for osteoblasts, and TRAP for osteoclasts^49^. A region of interest is defined in the trabecular bone in the metaphysis and BFR/bone surface (BS), MAR, BS, Ob.S/BS, and osteoclast surface (Oc.S/BS) are measured using a Nikon Optiphot 2 microscope interfaced to a semiautomatic analysis system (Osteometrics). Measurements were taken on two sections/sample (separated by ~25 μm) and summed prior to normalization to obtain a single measure/sample in accordance with ASBMR standards^50^. This methodology has undergone extensive quality control and validation and the results were assessed by two different researchers in a blinded fashion.

For immunofluorescence, fresh femurs and vertebrae dissected from rAAV-treated mice were collected and immediately fixed in ice-cold 4% paraformaldehyde solution for 2 days. Semidecalcification was carried out for 5 days in 0.5 M EDTA pH 7.4 at 4 °C with constant shaking (age ≥ 1 week), and infiltration was followed with a mixture of 20% sucrose phosphate buffer for 1 day and with 25% sucrose phosphate buffer next day. All samples were embedded in 50/50 mixture of 25% sucrose solution and OCT compound (Sakura) and cut into 12-μm-thick sagittal sections using a cryostat (Leica). Immunofluorescence staining and analysis was performed as described previously^49,51^. Briefly, after treatment with 0.2% Triton X-100 for 10 min, sections were blocked with 5% donkey serum at room temperature for 30 min and incubated overnight at 4 °C with anti-BGLAP antibody (sc-365797, Santa Cruz, 1:150). Primary antibodies were visualized with donkey anti-rat IgG Alexa-594 (1:500, Molecular Probes). Nuclei were counterstained with 4-6,diamidino-2-phenylindole (DAPI). An Olympus IX81 confocal microscope or Leica TCS SP5 II Zeiss LSM-880 confocal microscope was used to image samples.

### Biomechanical analysis

Femora were mechanically tested in three-point bending using an electrical force mechanical testing machine (Electroforce 3230, Bose Corporation, Eden Prairie, MN) at the Center for Skeletal Research Imaging and Biomechanical Testing Core. The bending fixture had a bottom span length of 8 mm. The test was performed with the load point in displacement control moving at a rate of 0.05 mm/s with force and displacement data collected at 60 Hz. All of the bones were positioned in the same orientation during testing with the cranial surface resting on the supports and being loaded in tension. Bending rigidity (EI, N-mm^2^), apparent modulus of elasticity (Eapp, MPa), ultimate moment (Mult, N-mm), apparent ultimate stress (σapp, MPa), work to fracture (Wfrac, mJ), and apparent toughness (Uapp, mJ/mm^3^) were calculated based on the force and displacement data from the tests and the mid-shaft geometry measured with microCT. Work to fracture is the energy that was required to cause the femur to fracture, and it was calculated by finding the area under the force–displacement curve using the Riemann Sum method. Bending rigidity was calculated using the linear portion of the force–displacement curve. The minimum moment of inertia was used when calculating the apparent modulus of elasticity.

### ELISA analysis

CTX1 ELISA (Abclonal MC0850) analysis was performed by using a kit according to the manufacturer’s instructions.

### Osteoblast differentiation analysis

For alkaline phosphatase (ALP) staining, osteoblasts were fixed with 10% neutral formalin buffer and stained with the solution containing Fast Blue (Sigma, FBS25) and Naphthol AS-MX (Sigma, 855). Alternatively, osteoblasts were incubated with tenfold diluted Alamar Blue solution (Invitrogen, DAL1100) for cell proliferation. Subsequently, cells were washed and incubated with a solution containing 6.5 mM Na_2_CO_3_, 18.5 mM NaHCO_3_, 2 mM MgCl_2_, and phosphatase substrate (Sigma, S0942), and ALP activity was measured by luminometer (Biorad).

To assess extracellular matrix mineralization in mature osteoblasts, cells were washed twice with phosphate-buffered saline (PBS) and fixed in 70% EtOH for 15 min at room temperature. Fixed cells were washed twice with distilled water and then stained with a 2% alizarin red solution (Sigma, A5533) for 5 min. Cells were then washed three times with distilled water and examined for the presence of calcium deposits. Mineralization was quantified by the acetic acid extraction method^52^.

### Quantitative RT-PCR analysis

Total RNA was purified from cells using QIAzol (QIAGEN) and cDNA was synthesized using the High-Capacity cDNA Reverse Transcription Kit from Applied Biosystems. Quantitative RT-PCR was performed using SYBR® Green PCR Master Mix (Bio-Rad) with CFX connect RT-PCR detection system (Bio-Rad). To measure *shn3* mRNA levels in bone tissues, after removal of bone marrow, tibias were snap-frozen in liquid nitrogen for 30 s and in turn homogenized in 1 ml of QIAzol for 1 min.

Alternatively, femurs and tibias dissected from rAAV9-treated mice were crushed in Hanks Balanced Salt Solution (Life Technologies) containing 10 mM HEPES (pH 7.2) (CellGro) and enzymatically digested with 2.5 mg/mL Collagenase A (Roche) and 1 unit/mL Dispase II (Roche) for 15 min at 37 °C under gentle agitation. The resulting cell suspensions were filtered (40 µm), washed using PBS (pH 7.2) containing 0.5% BSA (Fraction V) and 2 mM EDTA. After washing, cells were resuspended in PBS (pH 7.2) with 2 mM EDTA and 1 µg/mL DAPI (live/dead exclusion) and EGFP-expressing cells were sorted using a FACS Aria II SORP cell sorter (Becton Dickinson) at the University of Massachusetts Medical School with exclusion of DAPI^+^ cells and doublets. Total RNA was purified from cells using QIAzol. Primers used for PCR are described in the Supplementary Table 1.

### Immunoblotting analysis

Cells were lysed in TNT lysis buffer (50 mM Tris-HCl (pH 7.4), 150 mM NaCl, 1% Triton X-100, 1 mM EDTA, 1 mM EGTA, 50 mM NaF, 1 mM Na_3_VO_4_, 1 mM PMSF, and protease inhibitor cocktail (Sigma)) and protein amounts from cell lysates were measured using the DC protein assay (Bio-Rad). Equivalent amounts of proteins were subjected to SDS-PAGE, transferred to Immunobilon-P membranes (Millipore), immunoblotted with anti-GFP antibody (JL-8, 632381, Takara, 1:1000), anti-Cre recombinase antibody (ab24607, Abcam, 1:1000), anti-Hsp90 antibody (675402, Biolegend, 1:1000), and developed with ECL (Thermo fisher scientific). Immunoblotting with anti-HSP90 antibody was used as a loading control. Alternatively, dissected femurs and soft tissues were homogenized in RIPA lysis buffer (89900, Thermo fisher scientific) and tissue lysates were subjected to immunoblotting analysis.

### In vitro transduction assay of rAAV serotypes

ATDC5 cells or primary COBs were plated at a density of 1 × 10^4^ cells/well in 24-well plate and 24 h later, they were incubated with rAAV1, rAAV2, rAAV3, rAAV4, rAAV5, rAAV6, rAAV6.2, rAAV7, rAAV8, rAAV9, rAAVrh8, rAAVrh10, rAAVrh39, or rAAVrh43 vectors packaging the *CB-Egfp* reporter transgene at three different titers (10^9^–10^11^/mL genome copies). After 48 h, cells were washed with PBS and EGFP expression was monitored by the EVOS FL imaging system (Thermo fisher scientific). Alternatively, cells were lysed in TNT lysis buffer and EGFP expression was assessed by immunoblotting with anti-EGFP antibody and quantified using ImageJ software. (http://rsbweb,nih,gov/ij/). Lastly, primary bone marrow monocytes were plated at a density of 5 × 10^5^ cells/well in 24-well plates and cultured in the presence of 10 ng/ml of RAMKL and 20 ng/ml of M-CSF for 2 days to differentiate to osteoclast precursors. Three days after treatment with rAAV-*Egfp* vectors, EGFP expression was assessed by EVOS FL imaging system and by immunoblotting with anti-EGFP antibody.

For screening rAAV vectors in vivo, 10 μl of rAAV-*Egfp* vectors (1 × 10^11^ GC; 5 × 10^12^ GC/kg) was i.a. injected into knee joints of 2-month-old male mice (Jackson Laboratory, C57BL/6 J and BALB/cJ). Two weeks after injection, femurs and knee joints were dissected for IVIS-100 optical imaging or cryo-sectioning.

### rAAV9-mediated delivery of Cre recombinase or *amiR-shn3*

For a local delivery, 10 μl of rAAV9 carrying *amiR-ctrl* or *amiR-shn3* (1 × 10^11^ GC; 5 × 10^12^ GC/kg) was i.a. injected into knee joints of 2-month-old male mice (Jackson Laboratory, C57BL/6 J). Two months after injection, femurs were dissected for microCT analysis.

For a systemic delivery, 200 μl of rAAV9 carrying *Egfp*, *Cre*, *amiR-ctrl*, or *amiR-shn3* (4 × 10^11^ GC; 2 × 10^13^ GC/kg) was i.v. injected into mice (Jackson Laboratory, C57BL/6J) and 2 months later, mice were subcutaneously injected with calcein and alizarin-3-methyliminodiacetic acid at 6-day interval for dynamic histomorphometric analysis. Nonlabeled mice were used to monitor EGFP expression using the IVIS-100 optical imaging or cryosections.

### Effects of rAAV9-mediated silencing of *shn3* in osteoporosis

Mouse models of postmenopausal osteoporosis were generated by anesthetizing and bilaterally OVX 3-month-old female mice (Jackson Laboratory, C57BL/6J). Six weeks after the surgery, sham or OVX mice were i.v. injected with 200 μl of rAAV9 or rAAV9.DSS-Nter carrying *amiR-ctrl* or *amiR-shn3* (4 × 10^11^ GC; 2 × 10^13^ GC/kg). Mice were randomly divided into six groups with rAAV9 or rAAV9.DSS-Nter: sham + rAAV9-*amiR-ctrl*, OVX + rAAV9-*amiR-ctrl*, OVX + rAAV9-*amiR-shn3*, sham + rAAV9.DSS-Nter-*amiR-ctrl*, OVX + rAAV9.DSS-Nter-*amiR-ctrl*, OVX + rAAV9.DSS-Nter-*amiR-shn3*. Seven weeks after the injection, mice were subcutaneously injected with calcein and alizarin-3-methyliminodiacetic acid at 6-day intervals for dynamic histomorphometric analysis. Nonlabeled mice were used to monitor EGFP expression using the IVIS-100 optical imaging or frozen-sections.

### Statistical methods

All data were presented as the mean ± s.e.m. Sample sizes were calculated on the assumption that a 30% difference in the parameters measured would be considered biologically significant with an estimate of sigma of 10–20% of the expected mean. Alpha and Beta were set to the standard values of 0.05 and 0.8, respectively. No animals or samples were excluded from analysis, and animals were randomized to treatment versus control groups, where applicable. For relavant data analysis, where relevant, we first performed the Shapiro–Wilk normality test for checking normal distributions of the groups. If normality tests passed, two-tailed, unpaired Student’s *t*-test and if normality tests failed, and Mann–Whitney tests were used for the comparisons between two groups. For the comparisons of three or four groups, we used one-way ANOVA if normality tests passed, followed by Tukey’s multiple comparison test for all pairs of groups. If normality tests failed, Kruskal–Wallis test was performed and was followed by Dunn’s multiple comparison test. The GraphPad PRISM software (v6.0a, La Jolla, CA) was used for statistical analysis. *P* < 0.05 was considered statistically significant. *P < 0.05; **P < 0.01; ***P < 0.001; and ****P < 0.0001.

### Reporting summary

Further information on research design is available in the Nature Research Reporting Summary linked to this article.

## Supplementary information

Supplementary information

Reporting Summary

## Data Availability

Data supporting the findings of this manuscript are available from the corresponding author upon reasonable request.

## References

[1] Harada S, Rodan GA (2003). Control of osteoblast function and regulation of bone mass. Nature.

[2] Borumandi F, Aghaloo T, Cascarini L, Gaggl A, Fasanmade K (2015). Anti-resorptive drugs and their impact on maxillofacial bone among cancer patients. Anticancer Agents Med. Chem..

[3] Kraenzlin ME, Meier C (2011). Parathyroid hormone analogues in the treatment of osteoporosis. Nat. Rev. Endocrinol..

[4] Esbrit P, Alcaraz MJ (2013). Current perspectives on parathyroid hormone (PTH) and PTH-related protein (PTHrP) as bone anabolic therapies. Biochem. Pharm..

[5] Augustine M, Horwitz MJ (2013). Parathyroid hormone and parathyroid hormone-related protein analogs as therapies for osteoporosis. Curr. Osteoporos. Rep..

[6] MacNabb C, Patton D, Hayes JS (2016). Sclerostin antibody therapy for the treatment of osteoporosis: clinical prospects and challenges. J. Osteoporos..

[7] Bone HG (2010). Odanacatib, a cathepsin-K inhibitor for osteoporosis: a two-year study in postmenopausal women with low bone density. J. Bone Min. Res..

[8] Mullard A (2016). Merck & Co. drops osteoporosis drug odanacatib. Nat. Rev. Drug Discov..

[9] Jones DC (2006). Regulation of adult bone mass by the zinc finger adapter protein Schnurri-3. Science.

[10] Xu R (2018). Targeting skeletal endothelium to ameliorate bone loss. Nat. Med..

[11] Shim JH (2013). Schnurri-3 regulates ERK downstream of WNT signaling in osteoblasts. J. Clin. Investig.

[12] Wein M. N., Jones D. C., Shim J.-H., Aliprantis A. O., Sulyanto R., Lazarevic V., Poliachik S. L., Gross T. S., Glimcher L. H. (2012). Control of bone resorption in mice by Schnurri-3. Proceedings of the National Academy of Sciences.

[13] Rose JA, Hoggan MD, Shatkin AJ (1966). Nucleic acid from an adeno-associated virus: chemical and physical studies. Proc. Natl Acad. Sci. USA.

[14] Vandenberghe LH, Wilson JM, Gao G (2009). Tailoring the AAV vector capsid for gene therapy. Gene Ther..

[15] Snyder RO (1993). Features of the adeno-associated virus origin involved in substrate recognition by the viral Rep protein. J. Virol..

[16] McCarty DM, Ryan JH, Zolotukhin S, Zhou X, Muzyczka N (1994). Interaction of the adeno-associated virus Rep protein with a sequence within the A palindrome of the viral terminal repeat. J. Virol..

[17] McCarty DM (2003). Adeno-associated virus terminal repeat (TR) mutant generates self-complementary vectors to overcome the rate-limiting step to transduction in vivo. Gene Ther..

[18] Wang Z (2003). Rapid and highly efficient transduction by double-stranded adeno-associated virus vectors in vitro and in vivo. Gene Ther..

[19] Kyostio SR (1994). Analysis of adeno-associated virus (AAV) wild-type and mutant Rep proteins for their abilities to negatively regulate AAV p5 and p19 mRNA levels. J. Virol..

[20] Dubielzig R, King JA, Weger S, Kern A, Kleinschmidt JA (1999). Adeno-associated virus type 2 protein interactions: formation of pre-encapsidation complexes. J. Virol..

[21] Asokan A, Schaffer DV, Samulski RJ (2012). The AAV vector toolkit: poised at the clinical crossroads. Mol. Ther..

[22] Ulrich-Vinther M (2007). Gene therapy methods in bone and joint disorders. Evaluation of the adeno-associated virus vector in experimental models of articular cartilage disorders, periprosthetic osteolysis and bone healing. Acta Orthop. Suppl..

[23] Naso MF, Tomkowicz B, Perry WL, Strohl WR (2017). Adeno-associated virus (AAV) as a vector for gene therapy. BioDrugs.

[24] Zhang G (2012). A delivery system targeting bone formation surfaces to facilitate RNAi-based anabolic therapy. Nat. Med..

[25] Balakrishnan B, Jayandharan GR (2014). Basic biology of adeno-associated virus (AAV) vectors used in gene therapy. Curr. Gene Ther..

[26] Maes C (2010). Osteoblast precursors, but not mature osteoblasts, move into developing and fractured bones along with invading blood vessels. Dev. Cell.

[27] Muzumdar MD, Tasic B, Miyamichi K, Li L, Luo L (2007). A global double-fluorescent Cre reporter mouse. Genesis.

[28] Grimm D (2006). Fatality in mice due to oversaturation of cellular microRNA/short hairpin RNA pathways. Nature.

[29] McBride JL (2008). Artificial miRNAs mitigate shRNA-mediated toxicity in the brain: implications for the therapeutic development of RNAi. Proc. Natl Acad. Sci. USA.

[30] Xie, J. et al. A novel rAAV-amiRNA platform enables potent in vivo gene silencing and a ten-fold enhancement of on-target specificity over conventional shRNA vectors. in *American Society of Gene and Cell Therapy 21st Annual Meeting* (Chicago, IL., 2018).

[31] Bouxsein ML (2005). Ovariectomy-induced bone loss varies among inbred strains of mice. J. Bone Miner. Res..

[32] Saag KG (2017). Romosozumab or alendronate for fracture prevention in women with osteoporosis. N. Engl. J. Med..

[33] Wu P (2000). Mutational analysis of the adeno-associated virus type 2 (AAV2) capsid gene and construction of AAV2 vectors with altered tropism. J. Virol..

[34] Basner-Tschakarjan E, Mingozzi F (2014). Cell-mediated immunity to AAV vectors, evolving concepts and potential solutions. Front. Immunol..

[35] Zhong, L. et al. Development of novel recombinant AAV vectors and strategies for the potential gene therapy of hemophilia. *J. Genet. Syndr. Gene Ther.* **S1**, S1-008 (2012). 10.4172/2157-7412.S1-008 PMC3526190 23264889

[36] Gao G, Vandenberghe LH, Wilson JM (2005). New recombinant serotypes of AAV vectors. Curr. Gene Ther..

[37] Rivera VM (2005). Long-term pharmacologically regulated expression of erythropoietin in primates following AAV-mediated gene transfer. Blood.

[38] Yin H (2014). Non-viral vectors for gene-based therapy. Nat. Rev. Genet..

[39] Dayton RD, Wang DB, Klein RL (2012). The advent of AAV9 expands applications for brain and spinal cord gene delivery. Expert Opin. Biol. Ther..

[40] Foust KD (2009). Intravascular AAV9 preferentially targets neonatal neurons and adult astrocytes. Nat. Biotechnol..

[41] Mattar CN (2015). Systemic gene delivery following intravenous administration of AAV9 to fetal and neonatal mice and late-gestation nonhuman primates. FASEB J..

[42] Bish LT (2008). Adeno-associated virus (AAV) serotype 9 provides global cardiac gene transfer superior to AAV1, AAV6, AAV7, and AAV8 in the mouse and rat. Hum. Gene Ther..

[43] Luo F (2018). Adeno-associated virus-mediated RNAi against mutant alleles attenuates abnormal calvarial phenotypes in an apert syndrome mouse model. Mol. Ther. Nucleic Acids.

[44] Liang C (2015). Aptamer-functionalized lipid nanoparticles targeting osteoblasts as a novel RNA interference-based bone anabolic strategy. Nat. Med..

[45] Xie J (2017). Short DNA hairpins compromise recombinant adeno-associated virus genome homogeneity. Mol. Ther..

[46] Gao G, Sena-Esteves M (2012). Introducing genes into mammalian cells: viral vectors. Mol. Cloning.

[47] Jones D. C., Schweitzer M. N., Wein M., Sigrist K., Takagi T., Ishii S., Glimcher L. H. (2010). Uncoupling of growth plate maturation and bone formation in mice lacking both Schnurri-2 and Schnurri-3. Proceedings of the National Academy of Sciences.

[48] Park D (2012). Endogenous bone marrow MSCs are dynamic, fate-restricted participants in bone maintenance and regeneration. Cell Stem Cell.

[49] Fukuda T (2013). Sema3A regulates bone-mass accrual through sensory innervations. Nature.

[50] Parfitt AM (1987). Bone histomorphometry: standardization of nomenclature, symbols, and units. report of the ASBMR histomorphometry nomenclature committee. J. Bone Min. Res..

[51] Xu Ren, Zhang Chao, Shin Dong Yeon, Kim Jung-Min, Lalani Sarfaraz, Li Na, Yang Yeon-Suk, Liu Yifang, Eiseman Mark, Davis Roger J, Shim Jae-Hyuck, Greenblatt Matthew B (2017). c-Jun N-Terminal Kinases (JNKs) Are Critical Mediators of Osteoblast Activity In Vivo. Journal of Bone and Mineral Research.

[52] Gregory CA, Gunn WG, Peister A, Prockop DJ (2004). An Alizarin red-based assay of mineralization by adherent cells in culture: comparison with cetylpyridinium chloride extraction. Anal. Biochem..

