# Supplementary material for: Bone-targeting AAV-mediated silencing of Schnurri-3 prevents bone loss in osteoporosis
Source: Nat Commun. 2019 Jul 4;10:2958. doi: 10.1038/s41467-019-10809-6 (PMC6609711; doi:10.1038/s41467-019-10809-6)
Supplement: Supplementary file 1 — Supplementary information [file 41467_2019_10809_MOESM1_ESM.pdf]

Supplementary Information

Bone-targeting AAV-mediated silencing of Schnurri-3 prevents bone loss in osteoporosis

Yeon-Suk Yang[1+], Jun Xie[3, 4, 5+], Dan Wang[3,4], Jung-Min Kim[1], Phillip W.L. Tai[3,4], Ellen Gravallese[1], Guangping Gao[2,3, 4, 5*], and Jae-Hyuck Shim[1,2*]

[1]Department of Medicine/Division of Rheumatology, University of Massachusetts Medical School, Worcester, MA, USA

[2]Li Weibo Institute for Rare Diseases Research, University of Massachusetts Medical School, Worcester, MA, USA

[3]Horae Gene Therapy Center, [4]Department of Microbiology and Physiological Systems, [5]Viral Vector Core, University of Massachusetts Medical School, Worcester, MA, USA

+These authors contributed equally to this work.

*To whom correspondence should be addressed.

Jae-Hyuck Shim and Guangping Gao

Supplementary Table 1: Sequences of primers, probes and gblocks

Gene	Forward	Reverse
Mouse *shn3*	AGAGGCCATTCAGACGAGTGT	CTGCGGAAGCTGAGAGATGT
Mouse *Alp*	CACAATATCAAGGATATCGACGTGA	ACATCAGTTCTGTTCTTCGGGTACA
Mouse *Runx2*	TACAAACCATACCCAGTCCCTGTTT	AGTGCTCTAACCACAGTCCATGCA
Mouse *Bsp*	CAGGGAGGCAGTGACTCTTC	AGTGTGGAAAGTGTGGCGTT
Mouse *Osx*	ATGGCGTCCTCTCTGCTTGA	GAAGGGTGGGTAGTCATTTG
Mouse *Ocn*	GCAGCACAGGTCCTAAATAG	GGGCAATAAGGTAGTGAACAG
Mouse *Col1a1*	ACTGTCCCAACCCCCAAAG	ACGTATTCTTCCGGGCAGAA
Mouse *Hprt*	CTGGTGAAAAGGACCTCTCGAAG	CCAGTTTCACTAATGACACAAACG
EGFP	AGCAAAGACCCCAACGAGAA	GGCGGCGGTCACGAA
EGFP-probe	6FAM-CGCGATCACATGGTCCTGCTGG-TAMRA	
$(AspSerSer)_6$	GATTCATCAGATTCTTCTGATTCATCCGACTCTTCTGACAGTTCAGACAGCTCT	
amiR-33-ctrl (*amiR-ctrl*)	TTTGTCTTTTATTTCAGGTCCCAGATCTAGGGCTCTGCGTTTGCTCCAGGTAG<br>TCCGCTGCTCCCTTGGGCCTGGGCCCACTGACAGCCCTGGTGCCTCTGGCC<br>GGCTGCACACCTCCTGGCGGGCAGCTGTGTACAAACTACTTGAGAGCAGGT<br>GTTCTGGCAATACCTGCCTGCTCTGTAATAGTTTGTACACGGAGGCCTGCCC<br>TGACTGCCCACGGTGCCGTGGCCAAAGAGGATCTAAGGGCACCGCTGAGGG<br>CCTACCTAACCATCGTGGGGAATAAGGACAGTGTCACCCCTGCAGGGGATCC<br>GGTGGTGGTGCAAATCA	
amiR-33-SHN3 (*amiR-shn3*)	TTTGTCTTTTATTTCAGGTCCCAGATCTAGGGCTCTGCGTTTGCTCCAGGTAG<br>TCCGCTGCTCCCTTGGGCCTGGGCCCACTGACAGCCCTGGTGCCTCTGGCC<br>GGCTGCACACCTCCTGGCGGGCAGCTGTGTACAAACTACTTGAGAGCAGGT<br>GTTCTGGCAATACCTGCCTGCTCTGTAATAGTTTGTACACGGAGGCCTGCCC<br>TGACTGCCCACGGTGCCGTGGCCAAAGAGGATCTAAGGGCACCGCTGAGGG<br>CCTACCTAACCATCGTGGGGAATAAGGACAGTGTCACCCCTGCAGGGGATCC<br>GGTGGTGGTGCAAATCA	

Supplementary Figure. 1

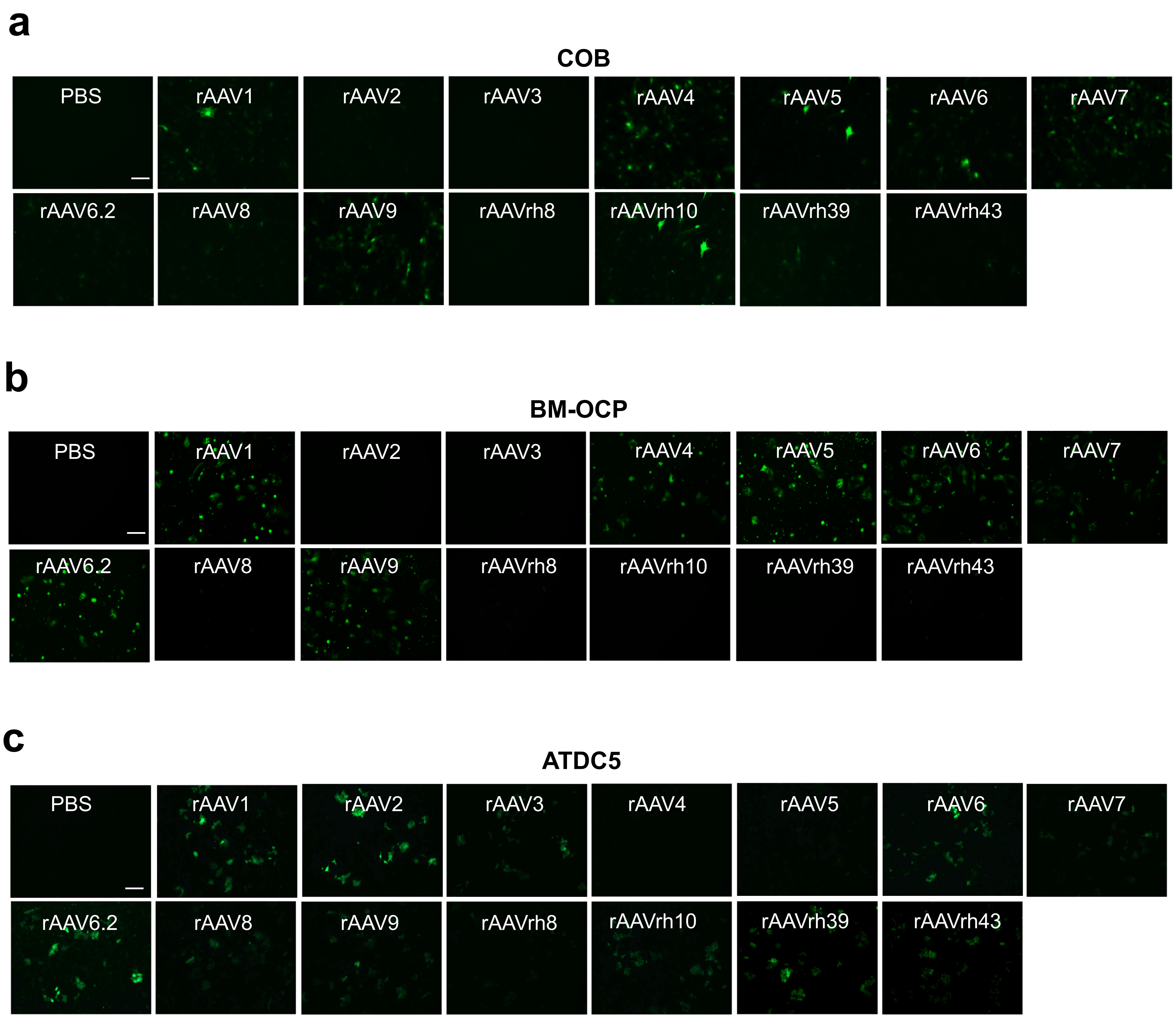

Supplementary Fig. 1: *In vitro* transduction assay of rAAV serotypes.
Calvarial osteoblasts (COB, **a**), bone marrow-derived osteoclast precursors (BM-OCP, **b**), or a chondrogenic cell line (ATDC5, **c**) were treated with PBS or 14 different AAV serotypes. Two days later, EGFP expression was monitored by fluorescence microscopy. Scale bar: 100 μm.

Supplementary Figure. 2

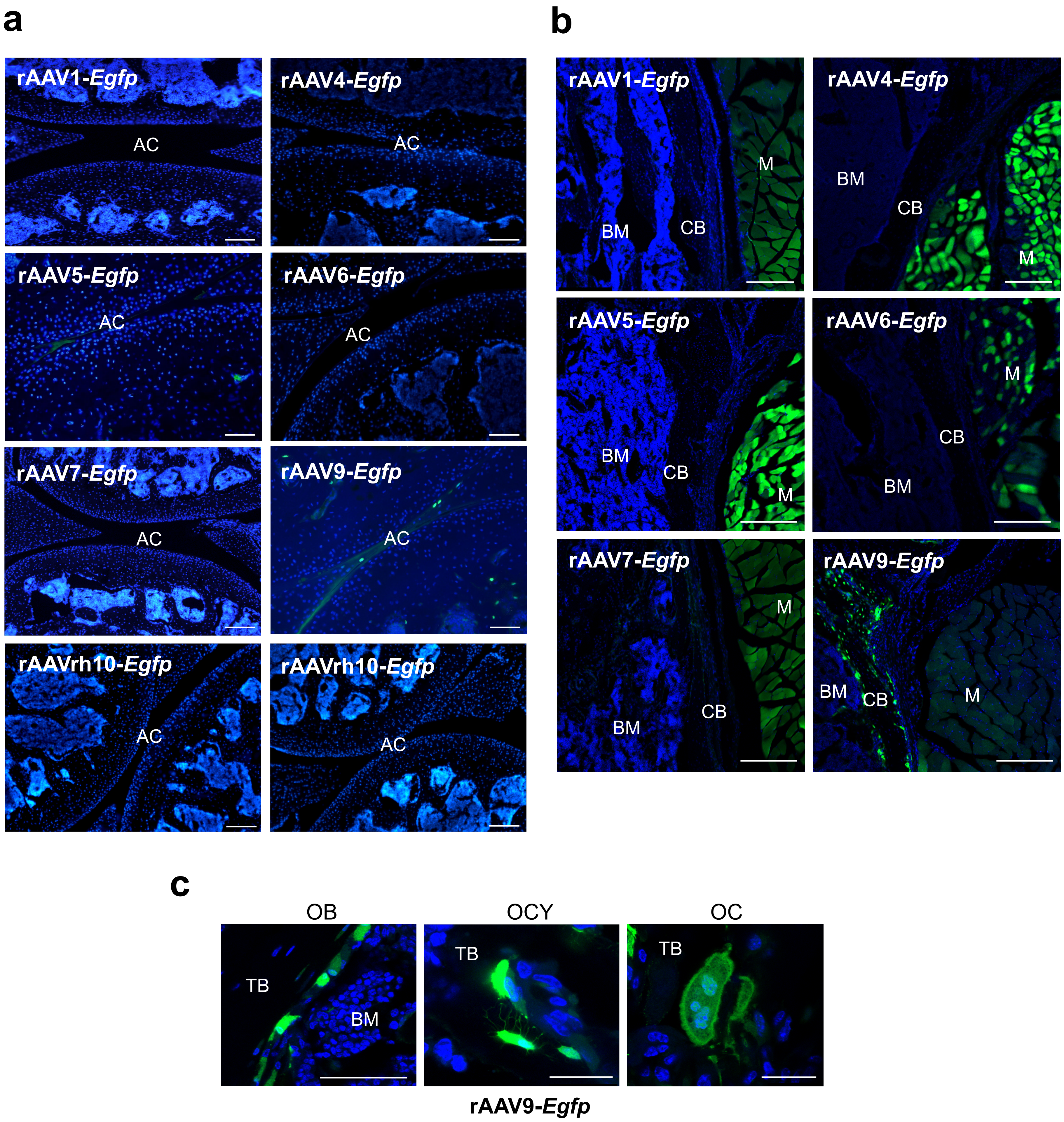

Supplementary Fig. 2: Identification of rAAV serotypes that transduce bone and cartilage. A single dose of PBS or 1 x 10^{11} genome copies of rAAV9-*Egfp* was intraarticularly (i.a.) injected into knee joints of two-month-old male mice. Knee joints **(a)** and femurs **(b)** were cryo-sectioned to identify EGFP-expressing cells. DAPI was used to stain nuclei. **Panel c** represents high-magnification images of EGFP-expressing osteoblasts (OB), osteocytes (OCY), and mature osteoclasts (OC). AC, articular cartilage; M, muscle; CB, cortical bone; BM, bone marrow. Scale bars: 100 μm, **panel a**; 250 μm, **panel b**; 75 μm (**left**) and 25 μm (**middle, right**), **panel c**.

Supplementary Figure. 3

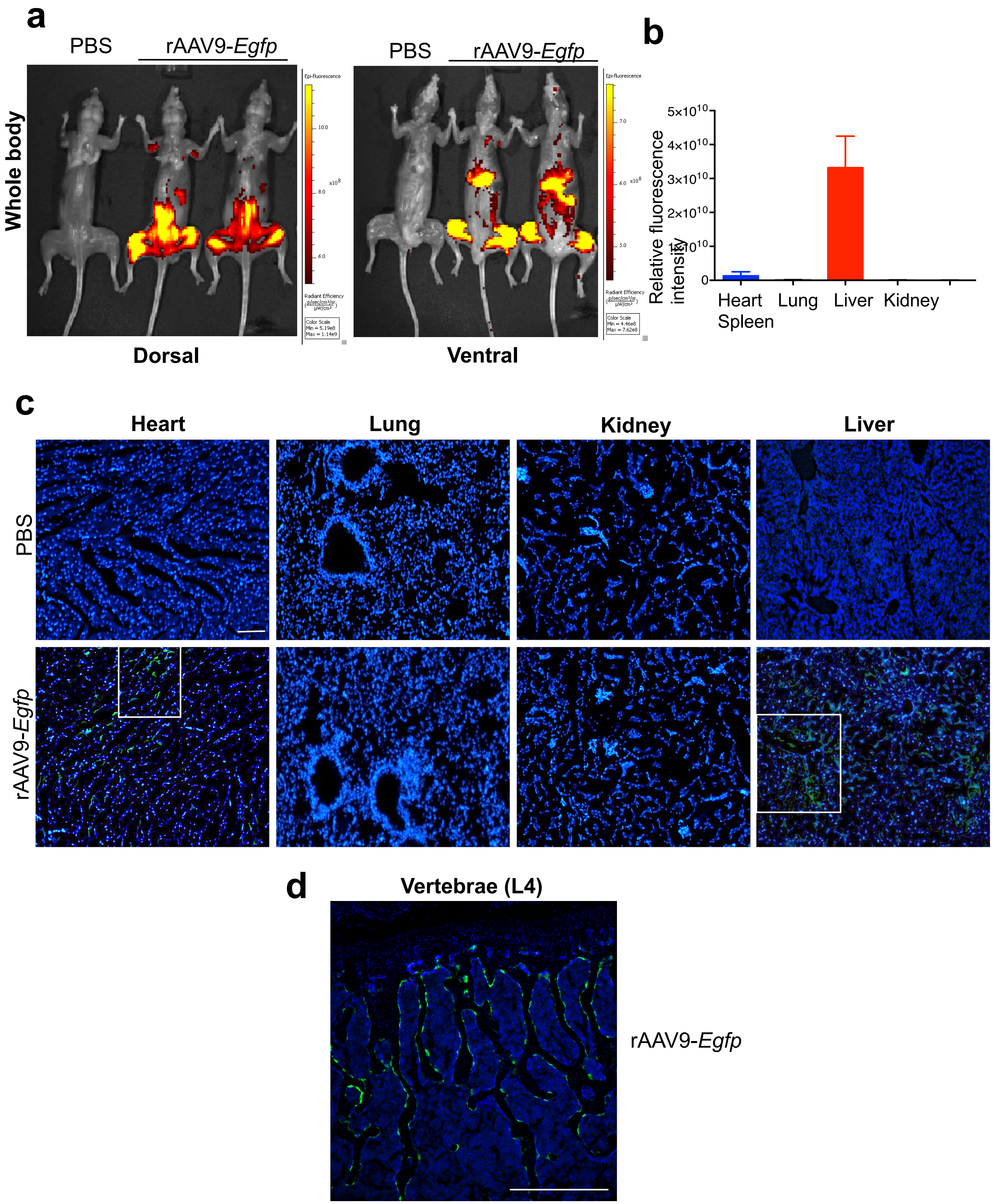

Supplementary Fig. 3: Tissue distribution of systemically-delivered rAAV9 in mice.
(**a, b**) A single dose of PBS or 4 x 10^{11} genome copies of rAAV9-*Egfp* was intravenously (i.v.) injected into two-month-old male mice and EGFP expression was monitored by IVIS-100 optical imaging two weeks post-injection. EGFP expression in whole body **(a)** and the quantification of EGFP expression in the dissected tissues shown in **Fig. 2a** are displayed **(b)**. PBS-injection was used as a negative control.
(**c, d**) Tissues dissected from PBS- or rAAV9-*Egfp*-injected mice were cryo-sectioned to locate EGFP-expressing cells. Scale bars: 100 μm, **panel c**; 500 μm, **panel d**.

Supplementary Figure. 4

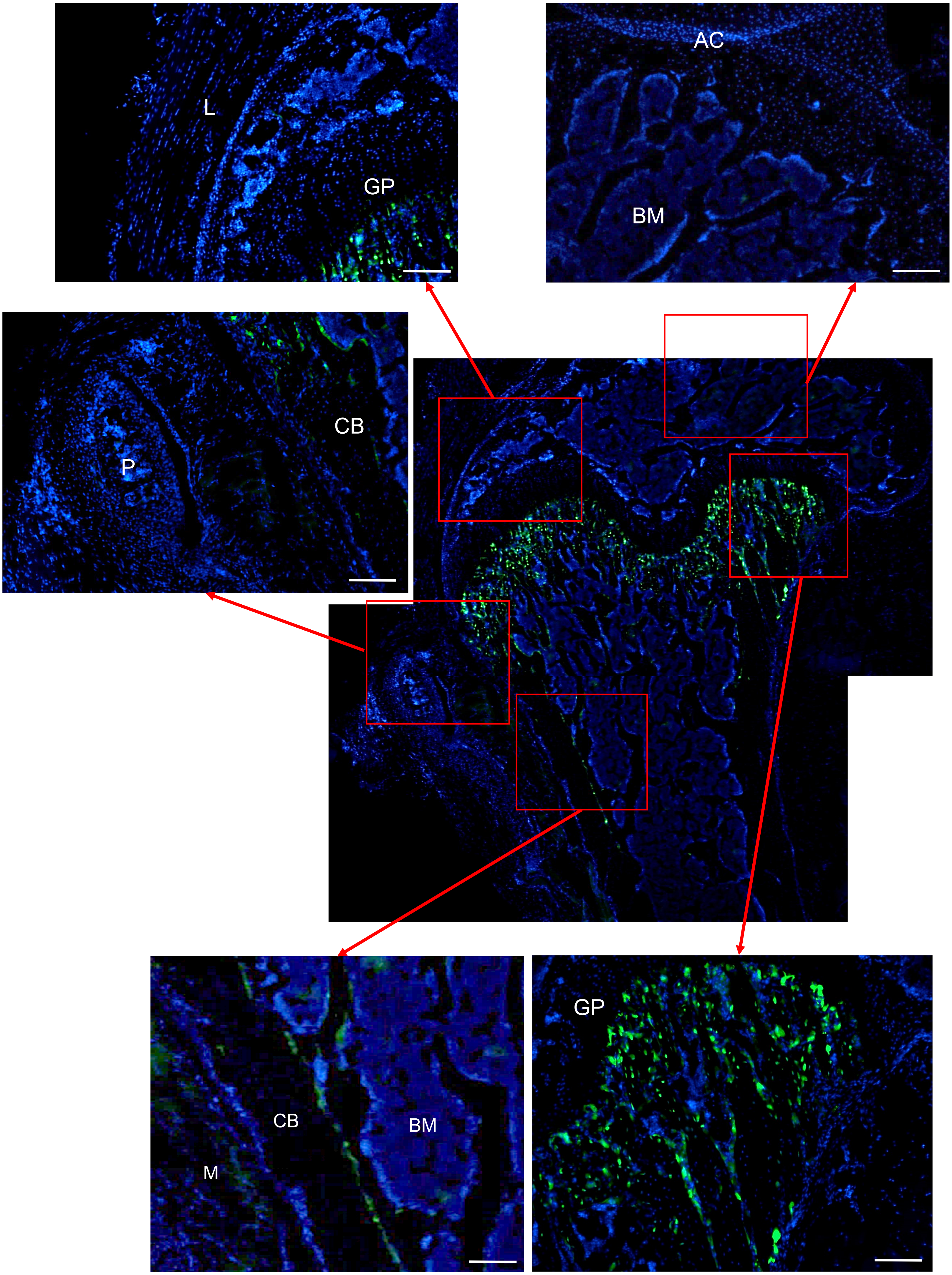

Supplementary Fig. 4: Distribution of systemically-delivered rAAV9 in the femur.
A single dose of 4 x 10^{11} genome copies of rAAV9-*Egfp* was i.v. injected into two-month-old male mice and EGFP expression was assessed in the cryo-sectioned femurs two weeks post-injection. L, Ligament; GP, growth plate; AC, articular cartilage; BM, bone marrow; CB, cortical bone; P, patella; M, muscle. Scale bars: 100 μm.

Supplementary Figure. 5

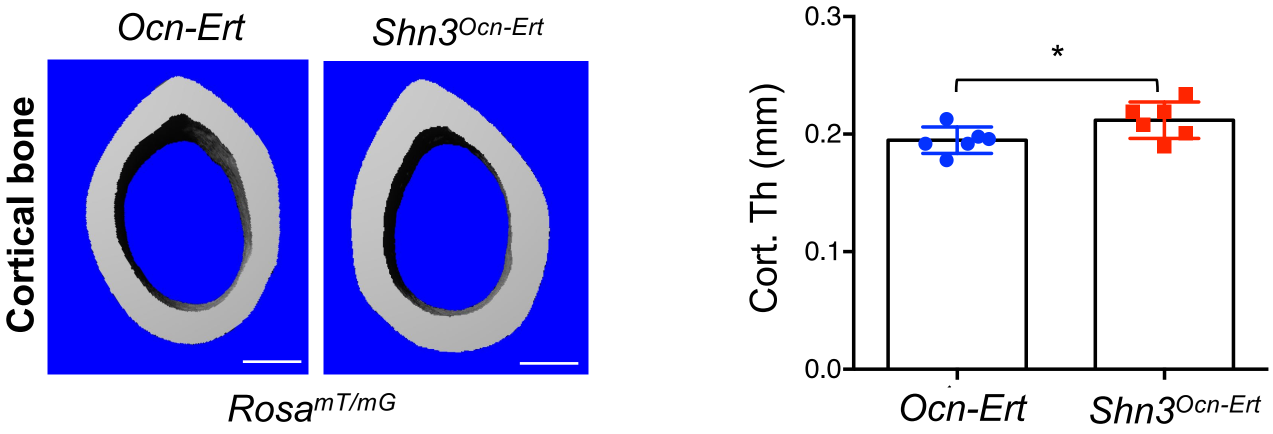

Supplementary Fig. 5: Inducible deletion of *Shn3* increases bone accrual in adult mice.
Two-month-old female *Ocn-Ert;Rosa*$^{mT/mG}$ and *Shn3*$^{Ocn\text{-}Ert}$*;Rosa*$^{mT/mG}$ mice were treated with 100 mg/kg of tamoxifen for five consecutive days; two months later, femoral cortical bone mass was assessed by microCT. Representative 3D-reconstruction **(left)** and relative quantification **(right)** are displayed. Cortical Thickness (Cort.Th) (n = 6/group). Values represent mean ± SD: *, $P < 0.05$ by an unpaired two-tailed Student's t-test. Scale bars: 1 mm.

Supplementary Figure. 6

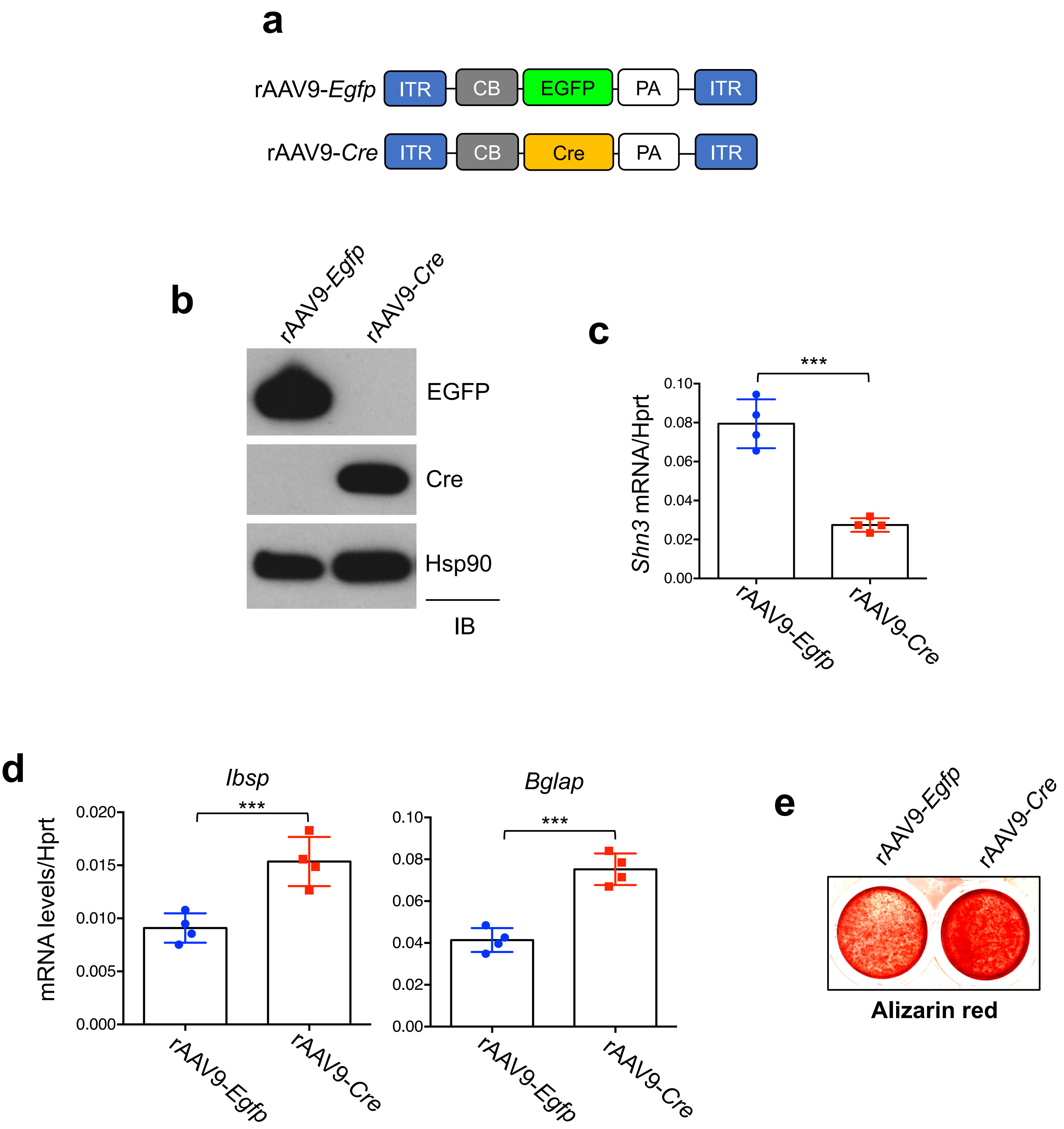

Supplementary Fig. 6: *In vitro* characterization of rAAV9-*Cre* in *Shn3*$^{fl/fl}$ osteoblasts.
(**a**) Diagram of rAAV9 constructs containing the CMV enhancer/chicken β-actin promoter (CB), an *Egfp* reporter gene (EGFP), or Cre recombinase (Cre), *β-globin* polyA sequence (PA), and inverted terminal repeat (ITR).
(**b**) COBs were infected with rAAV9-*Egfp* or rAAV9-*Cre* for two days and cells were lysed and immunoblotted with the indicated antibodies.
(**c-e**) Two days after treatment with rAAV9-*Egfp* or rAAV9-*Cre*, COBs were cultured under osteogenic conditions for six days and mRNA levels of *Shn3* **(c)** and osteogenic genes **(d)** were measured by RT-PCR. After 21 days of the culture, mineralization was assessed by alizarin red staining **(e)**. Values represent mean ± SD: ***, P < 0.001 by an unpaired two-tailed Student's t-test **(c, d)**.

Supplementary Figure. 7

a

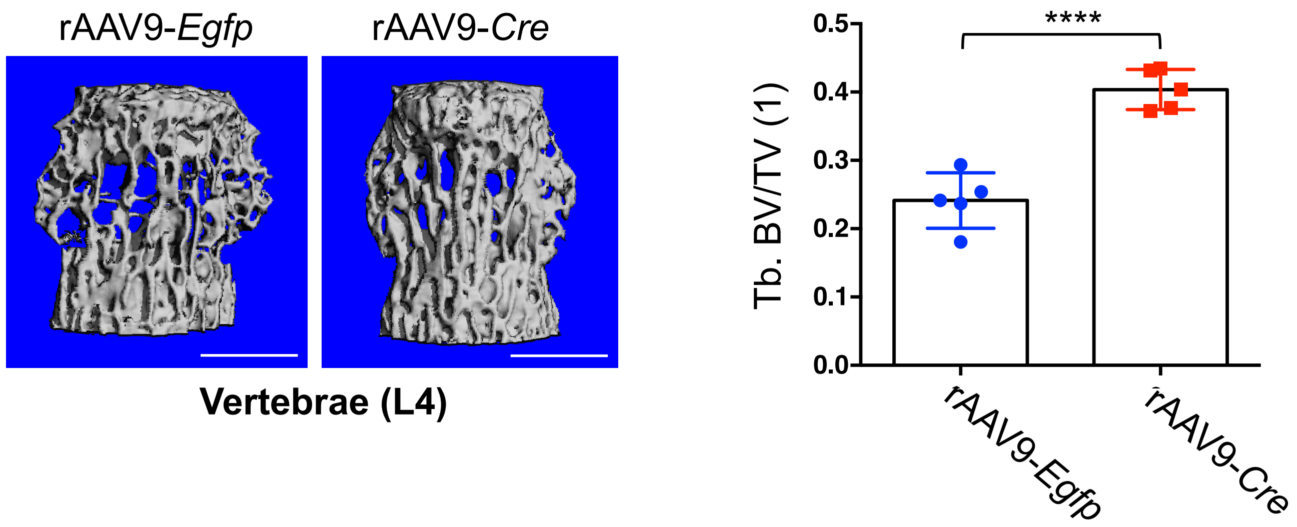

b

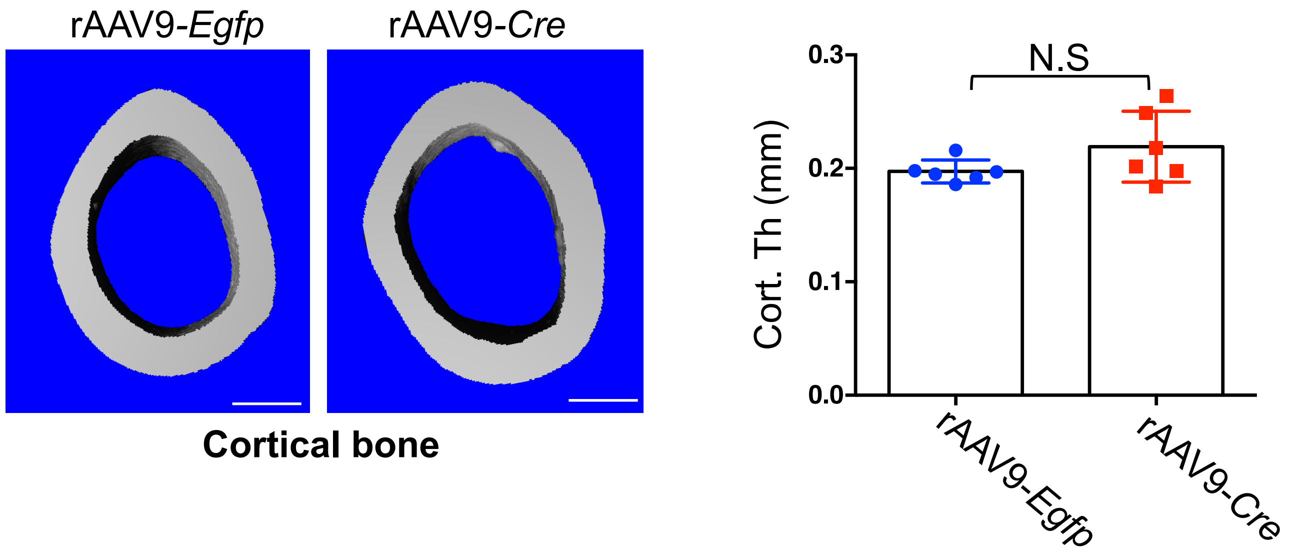

Supplementary Fig. 7: *In vivo* characterization of rAAV9-*Cre* in *Shn3*$^{fl/fl}$ mice.
A single dose of 4 x 10^{11} genome copies of rAAV9-*Egfp* or rAAV9-*Cre* was i.v. injected into three-month-old male *Shn3*$^{fl/fl}$;*Rosa*^{mTmG} mice. Two months later, trabecular bone mass in the lumbar vertebrae **(a)** and cortical bone thickness in the femur **(b)** were assessed by microCT. Representative 3D-reconstruction **(left)** and relative quantification **(right)** are displayed. Scale bars: 1 mm.

Supplementary Figure. 8

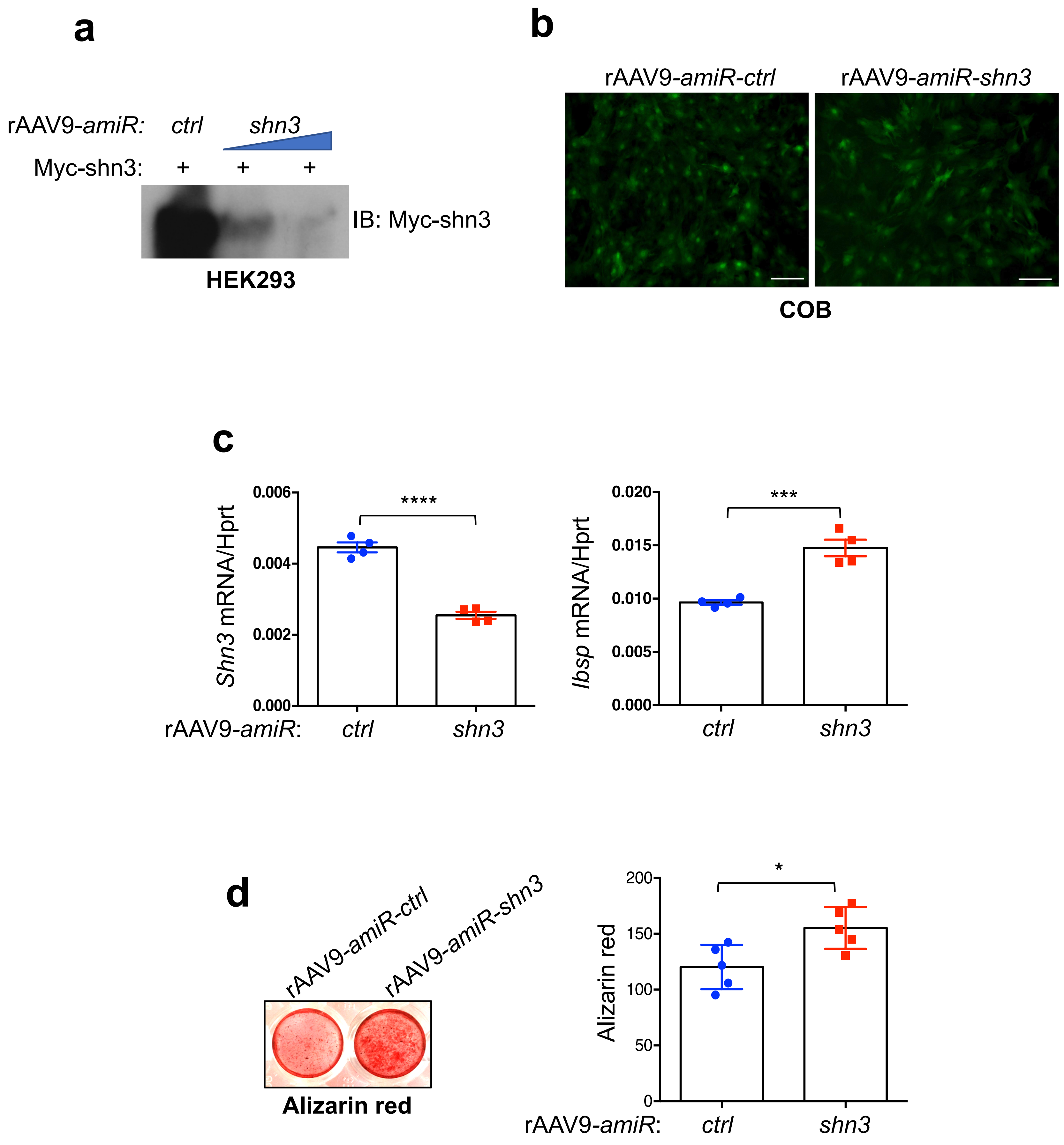

Supplementary Fig. 8: *In vitro* characterization of rAA9-*amiR-shn3* in osteoblasts.
(a) HEK293 cells were transiently transfected with Myc-tagged mouse *shn3* along with plasmids encoding *amiR-shn3* or *amiR-ctrl*. Two days later, cell lysates were immunoblotted with anti-Myc antibody.
(b-d) Two days after treatment with rAAV9 carrying *amiR-ctrl* or *amiR-shn3*, COBs were cultured under osteogenic conditions. EGFP expression **(b)**, mRNA levels of *Shn3* and osteogenic gene **(c)**, and mineralization **(d),** were assessed by fluorescence microscopy, RT-PCR, and alizarin red staining, respectively. Scale bar: 100 μm, **panel b**. Values represent mean ± SD: *, $P < 0.05$; ***, $P < 0.001$; and ****, $P < 0.0001$ by an unpaired two-tailed Student's t-test **(c, d)**.

Supplementary Figure. 9

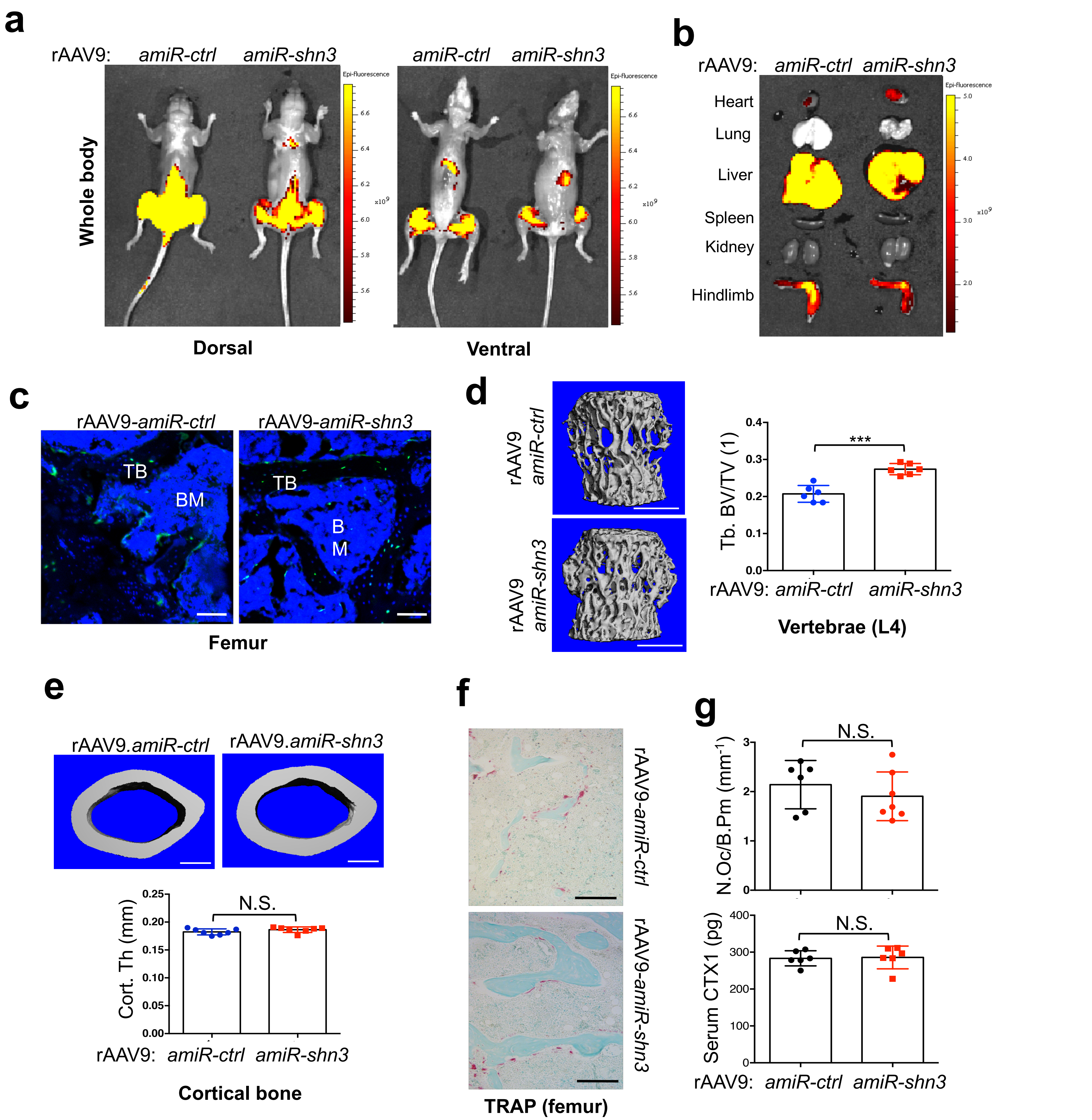

Supplementary Fig. 9: Characterize mice with systemically delivered rAAV9-*amiR-shn3.*
A single dose of 4 x 10^{11} genome copies of rAAV9 carrying *amiR-ctrl* or *amiR-shn3* was i.v. injected into three-month-old female mice. Two months following treatment, mice were labeled with calcein and alizarin red for dynamic histomorphometry. Non-labeled mice were used to monitor EGFP expression using IVIS-100 optical imaging. EGFP expression in the whole body **(a)** and the dissected tissues **(b)** are displayed. Femurs were cryo-sectioned to identify EGFP-expressing osteoblast lineage cells **(c)**. Trabecular bone mass in the lumbar vertebrae **(d)** and cortical bone thickness in the femur **(e)** were assessed by microCT. TRAP-stained longitudinal sections **(f)** and histomorphometric analysis **(top, g)** of femurs from five-month-old female mice treated with rAAV9 carrying *amiR-ctrl* or *amiR-shn3* (n = 6~7). The number of osteoclasts per bone perimeter (N.Oc/B.Pm). Serum CTX levels were assessed by ELISA (n = 6) **(bottom, g)**. Scale bars: 100 μm, **panel c**; 1 mm, **panel d and e**; 50 μm, **panel f**. Values represent mean ± SD; N.S., not significant; ***, P < 0.001 by an unpaired two-tailed Student's *t*-test **(d, e, g)**.

Supplementary Figure. 10

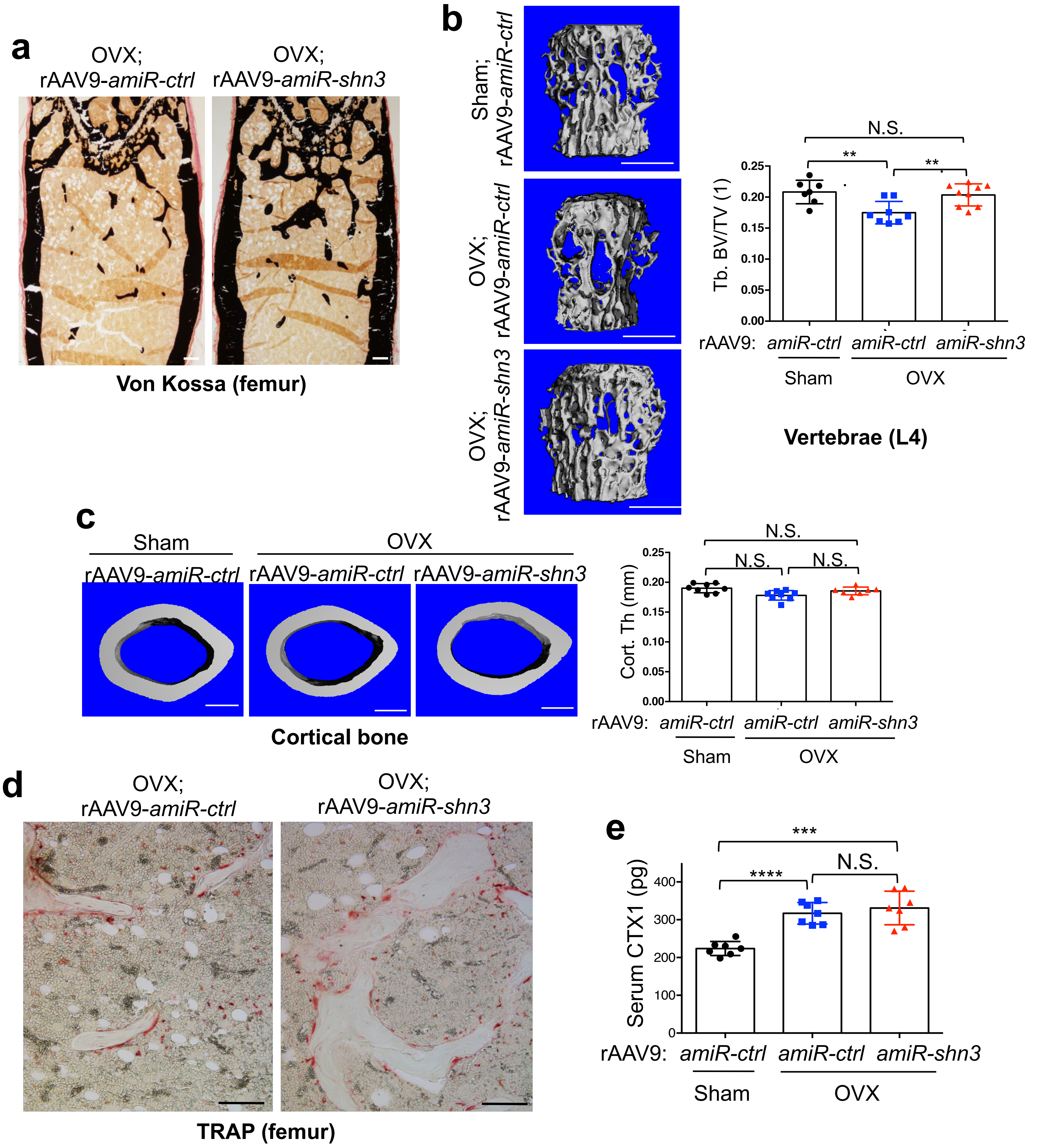

Supplementary Fig. 10: Effects of rAAV9-mediated silencing of *shn3* in osteoporosis.
Sham or OVX surgery was performed on three-month-old female mice and six weeks later, a single dose of 4 x 10^{11} genome copies of rAAV9 carrying *amiR-ctrl* or *amiR-shn3* was i.v. injected. Seven weeks after injection, mice were labeled with calcein and alizarin red for dynamic histomorphometry. Staining by Von Kossa **(a)** or TRAP **(d)** was performed in longitudinal sections of femurs from five-month-old female OVX mice treated with rAAV9 carrying *amiR-ctrl* or *amiR-shn3*. Trabecular bone mass in the lumbar vertebrae **(b)** and cortical bone thickness in the femur **(c)** were assessed by microCT. Serum CTX levels were assessed by ELISA (n = 7) **(e)**. Scale bar: 1 mm, **panels a-c**; 50 μm, **panel d**. Values represent mean ± SD; NS, not significant, **, P < 0.01; ***, *P* < 0.001 and ****, *P* < 0.0001 by an unpaired two-tailed Student's *t*-test **(c)**.

Supplementary Figure. 11

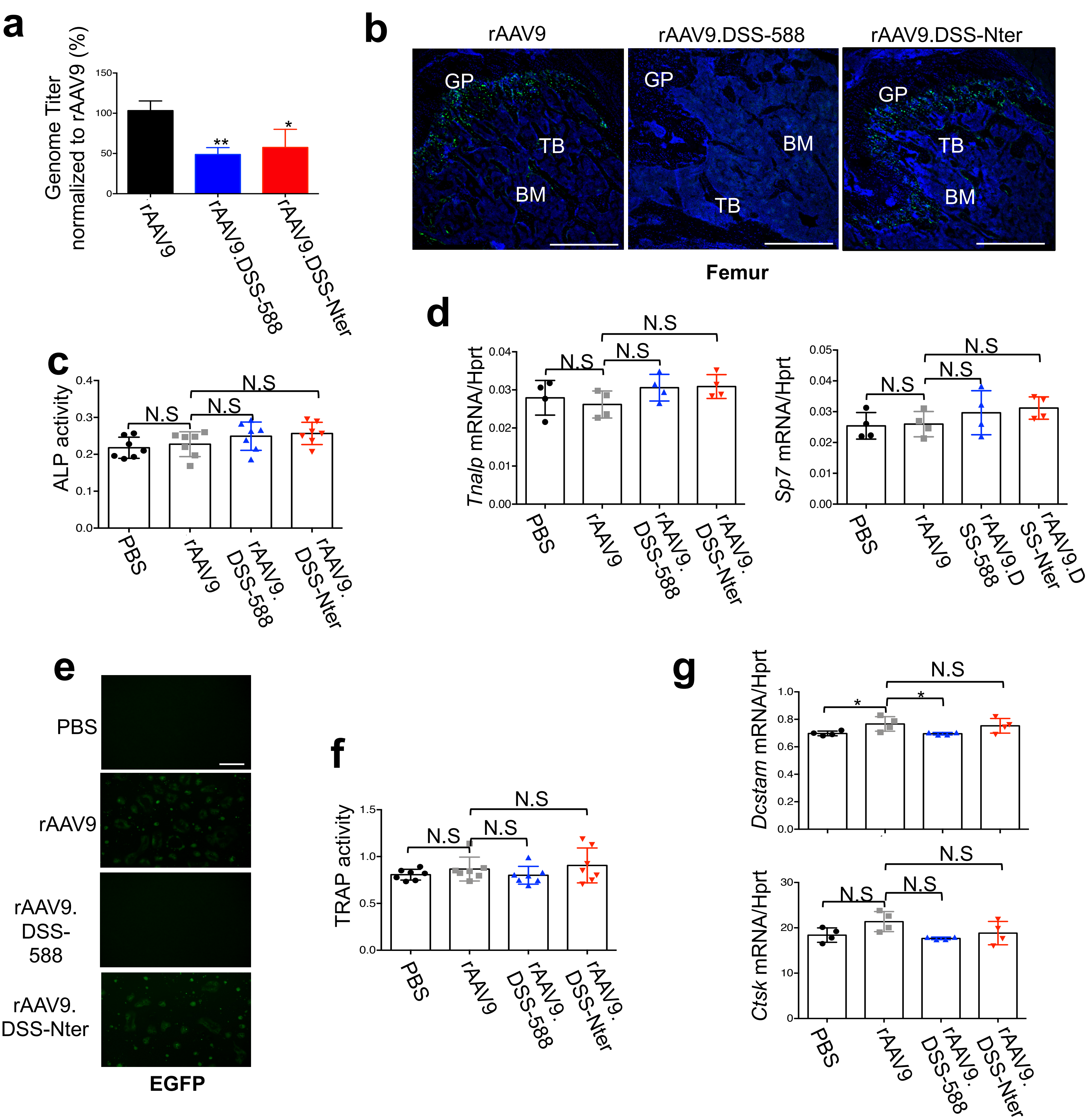

Supplementary Fig. 11: Characterization of rAAV9.DSS vectors *in vitro* and *in vivo*.
(**a**) The genome titers of rAAV9.DSS-588 and rAAV9.DSS-Nter were measured by ddPCR and normalized to that of rAAV9.
(b) A single dose of 1 x 10^{11} genome copies of rAAV9-*Egfp*, rAAV9.DSS-588-*Egfp* or rAAV9.DSS-Nter-*Egfp* was i.a. injected into knee joints of two-month-old male mice and femurs were cryo-sectioned to identify EGFP-expressing cells two weeks post-injection. GP, growth plate; CB, cortical bone; BM, bone marrow. Scale bar: 500 μm, **panel b**.
(c, d) Two days after infection with rAAV9-*Egfp*, rAAV9.DSS-588-*Egfp* or rAAV9.DSS-Nter-*Egfp*, COBs were cultured under osteogenic conditions for six days. Osteoblast differentiation was assessed by ALP activity **(c)** and osteogenic gene expression **(d)**.
(e-g) Two days after treatment with M-CSF (20 ng/ml) and Rank ligand (10 ng/ml), bone marrow-derived monocytes were incubated with rAAV9-*Egfp*, rAAV9.DSS-588-*Egfp* or rAAV9.DSS-Nter-*Egfp* and EGFP expression was monitored by fluorescence microscopy two days post-infection **(e)**. Osteoclast differentiation was assessed by TRAP activity **(f)** and osteoclast gene expression **(g)**. Values represent mean ± SD: N.S, non-significant; *, P < 0.05 by one-way ANOVA test. Scale bars: 500 μm, **panel b**; 1 mm, **panel e**.

Supplementary Figure. 12

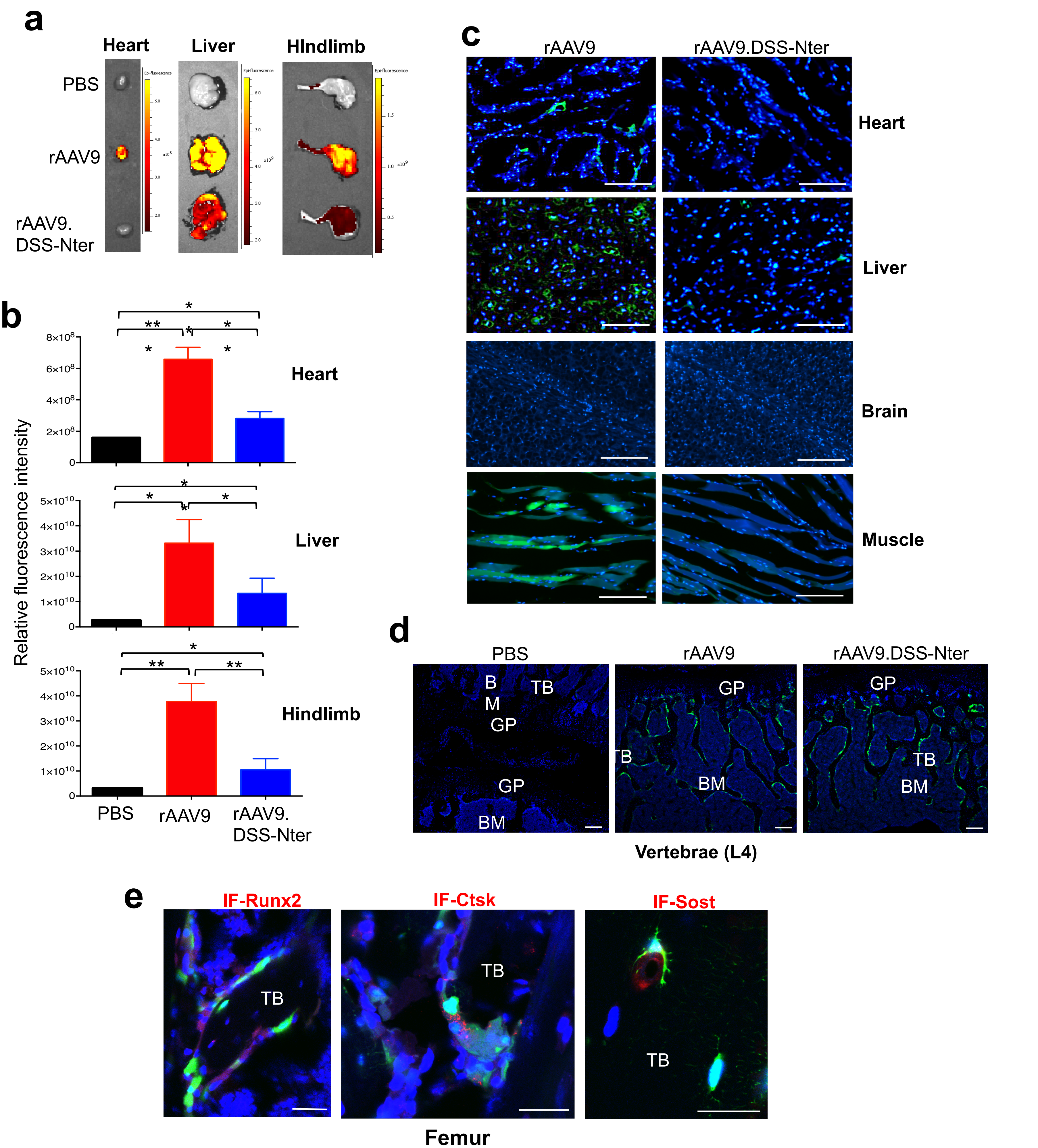

Supplementary Fig. 12: Tissue distribution of systemically delivered rAAV9.DSS-Nter.
A single dose of PBS or 4 x 10^{11} genome copies of rAAV9, rAAV9.DSS-588, or rAAV9.DSS-Nter was i.v. injected into two-month-old male mice and EGFP expression was monitored using IVIS-100 optical imaging two weeks post-injection. EGFP expression in the heart, liver, and hindlimb **(a)** and quantification of EGFP expression in dissected tissues **(b)** are displayed. Heart, liver, brain, muscle **(c)** and lumbar vertebrae **(d)** were cryo-sectioned to identify EGFP-expressing cells. Cryo-sectioned femurs were also immunostained for Runx2, Ctsk, and Sost to identify osteoblasts, mature osteoclasts, and osteocytes, respectively **(e).** GP, growth plate; BM, bone marrow; TB, trabecular bone. Scale bar: 100 μm, **panels c and d**; 25 μm, **panel e**. Values represent mean ± SD: *, $P < 0.05$; **, $P < 0.01$ and ***, $P < 0.001$ by one-way ANOVA test **(b)**.

Supplementary Figure. 13

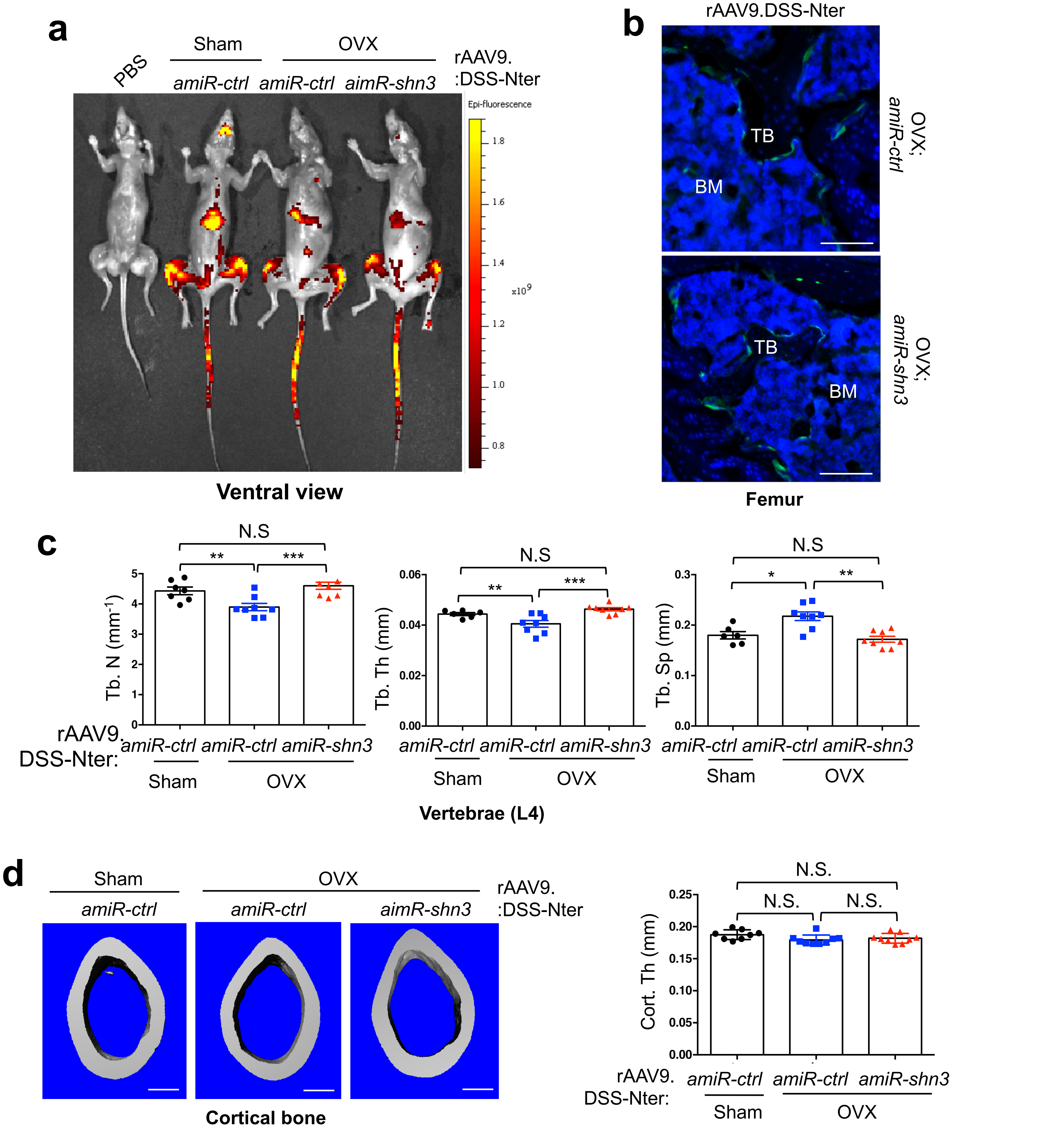

Supplementary Fig. 13: Effects of rAAV9.DSS-Nter carrying *amiR-shn3* in osteoporosis.
Sham or OVX surgery was performed on three-month-old female mice and six weeks later, a single dose of PBS or 4 x 10^{11} genome copies of rAAV9.DSS-Nter carrying *amiR-ctrl* or *amiR-shn3* were i.v. injected. Seven weeks after injection, EGFP expression was monitored by IVIS-100 optical imaging **(a)**. EGFP-expressing cells in the cryo-sectioned femurs were identified by fluorescence microscopy **(b)**. Trabecular bone mass in lumbar vertebrae **(c)** and cortical bone thickness in the femur **(d)** was assessed by microCT (n = 7~8/group). Trabecular number per cubic millimeter (Tb.N), trabecular thickness (Tb.Th), and trabecular space (Tb. Sp). TB, trabecular bone; BM, bone marrow. Scale bar: 100 μm, **panel b**. Values represent mean ± SD: N.S, non-significant; *, $P < 0.05$; **, $P < 0.01$; ***, $P < 0.001$ by one-way ANOVA test **(c)**.

Full uncut gel for Figure 1a

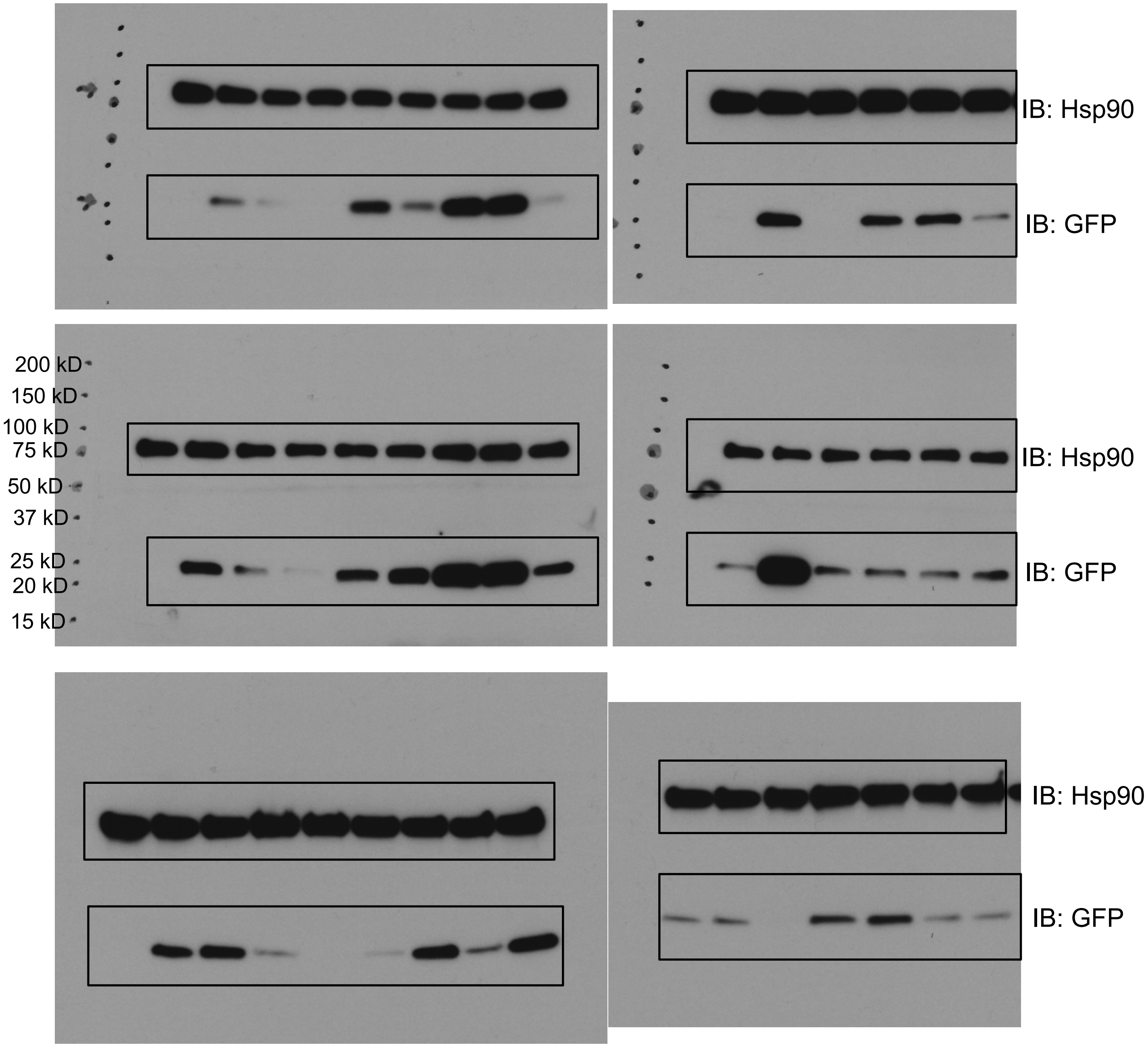

Full uncut gel for Figure 2d

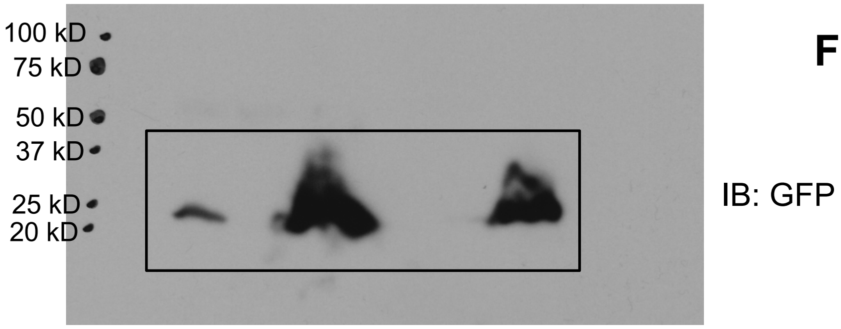

Full uncut gel for Figure 3d

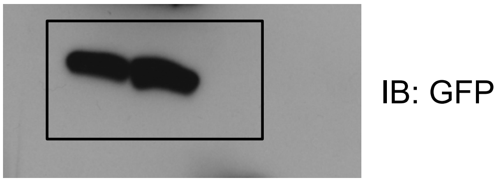

Full uncut gel for Figure 5b

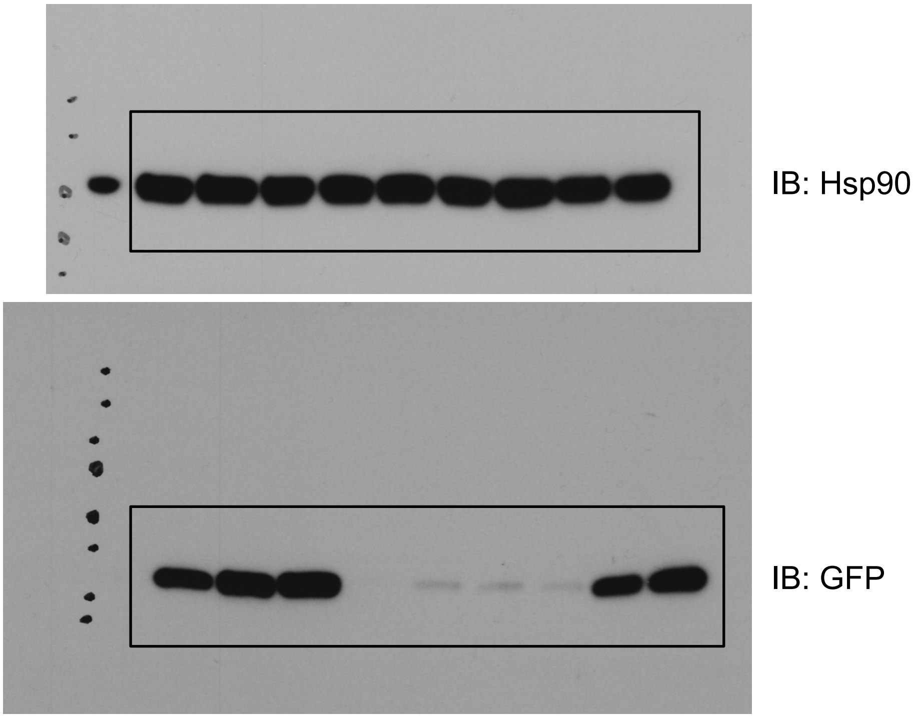

Full uncut gel for Figure 5e

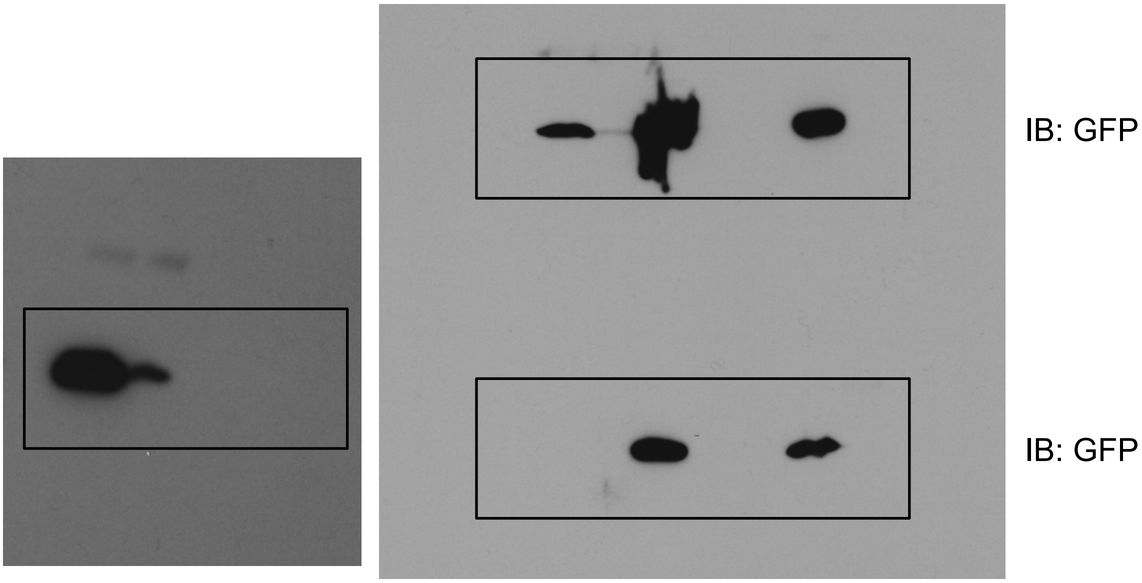

Supplementary Table 1: Sequences of primers, probes and gblocks

Gene	Forward	Reverse
Mouse *shn3*	AGAGGCCATTCAGACGAGTGT	CTGCGGAAGCTGAGAGATGT
Mouse *Alp*	CACAATATCAAGGATATCGACGTGA	ACATCAGTTCTGTTCTTCGGGTACA
Mouse *Runx2*	TACAAACCATACCCAGTCCCTGTTT	AGTGCTCTAACCACAGTCCATGCA
Mouse *Bsp*	CAGGGAGGCAGTGACTCTTC	AGTGTGGAAAGTGTGGCGTT
Mouse *Osx*	ATGGCGTCCTCTCTGCTTGA	GAAGGGTGGGTAGTCATTTG
Mouse *Ocn*	GCAGCACAGGTCCTAAATAG	GGGCAATAAGGTAGTGAACAG
Mouse *Col1a1*	ACTGTCCCAACCCCCAAAG	ACGTATTCTTCCGGGCAGAA
Mouse *Hprt*	CTGGTGAAAAGGACCTCTCGAAG	CCAGTTTCACTAATGACACAAACG
EGFP	AGCAAAGACCCCAACGAGAA	GGCGGCGGTCACGAA
EGFP-probe	6FAM-CGCGATCACATGGTCCTGCTGG-TAMRA	
(AspSerSer)$_6$	GATTCATCAGATTCTTCTGATTCATCCGACTCTTCTGACAGTTCAGACAGCTCT	
amiR-33-ctrl (*amiR-ctrl*)	TTTGTCTTTTATTTCAGGTCCCAGATCTAGGGCTCTGCGTTTGCTCCAGGTAG<br>TCCGCTGCTCCCTTGGGCCTGGGCCCACTGACAGCCCTGGTGCCTCTGGCC<br>GGCTGCACACCTCCTGGCGGGCAGCTGTGTACAAACTACTTGAGAGCAGGT<br>GTTCTGGCAATACCTGCCTGCTCTGTAATAGTTTGTACACGGAGGCCTGCCC<br>TGACTGCCCACGGTGCCGTGGCCAAAGAGGATCTAAGGGCACCGCTGAGGG<br>CCTACCTAACCATCGTGGGGAATAAGGACAGTGTCACCCCTGCAGGGGATCC<br>GGTGGTGGTGCAAATCA	
amiR-33-SHN3 (*amiR-shn3*)	TTTGTCTTTTATTTCAGGTCCCAGATCTAGGGCTCTGCGTTTGCTCCAGGTAG<br>TCCGCTGCTCCCTTGGGCCTGGGCCCACTGACAGCCCTGGTGCCTCTGGCC<br>GGCTGCACACCTCCTGGCGGGCAGCTGTGTACAAACTACTTGAGAGCAGGT<br>GTTCTGGCAATACCTGCCTGCTCTGTAATAGTTTGTACACGGAGGCCTGCCC<br>TGACTGCCCACGGTGCCGTGGCCAAAGAGGATCTAAGGGCACCGCTGAGGG<br>CCTACCTAACCATCGTGGGGAATAAGGACAGTGTCACCCCTGCAGGGGATCC<br>GGTGGTGGTGCAAATCA	
