# Supplementary material for: Bone-targeting AAV-mediated silencing of Schnurri-3 prevents bone loss in osteoporosis
Source: Nat Commun. 2019 Jul 4;10:2958. doi: 10.1038/s41467-019-10809-6 (PMC6609711; doi:10.1038/s41467-019-10809-6)
Supplement: Supplementary file 2 — Reporting Summary [file 41467_2019_10809_MOESM2_ESM.pdf]

nature research

Corresponding author(s): Jae-Hyuck Shim

Reporting Summary

Nature Research wishes to improve the reproducibility of the work that we publish. This form provides structure for consistency and transparency in reporting. For further information on Nature Research policies, see Authors & Referees and the Editorial Policy Checklist.

Statistical parameters

When statistical analyses are reported, confirm that the following items are present in the relevant location (e.g. figure legend, table legend, main text, or Methods section).

n/a	Confirmed	
☐	☒	The exact sample size (n) for each experimental group/condition, given as a discrete number and unit of measurement
☐	☒	An indication of whether measurements were taken from distinct samples or whether the same sample was measured repeatedly
☐	☒	The statistical test(s) used AND whether they are one- or two-sided<br>*Only common tests should be described solely by name; describe more complex techniques in the Methods section.*
☐	☒	A description of all covariates tested
☐	☒	A description of any assumptions or corrections, such as tests of normality and adjustment for multiple comparisons
☐	☒	A full description of the statistics including central tendency (e.g. means) or other basic estimates (e.g. regression coefficient) AND variation (e.g. standard deviation) or associated estimates of uncertainty (e.g. confidence intervals)
☐	☒	For null hypothesis testing, the test statistic (e.g. F, t, r) with confidence intervals, effect sizes, degrees of freedom and P value noted<br>*Give P values as exact values whenever suitable.*
☒	☐	For Bayesian analysis, information on the choice of priors and Markov chain Monte Carlo settings
☒	☐	For hierarchical and complex designs, identification of the appropriate level for tests and full reporting of outcomes
☐	☒	Estimates of effect sizes (e.g. Cohen's d, Pearson's r), indicating how they were calculated
☐	☒	Clearly defined error bars<br>*State explicitly what error bars represent (e.g. SD, SE, CI)*

Our web collection on statistics for biologists may be useful.

Software and code

Policy information about availability of computer code

Data collection	n/a
Data analysis	The GraphPad PRISM software (v6.0a, La Jolla, CA)

For manuscripts utilizing custom algorithms or software that are central to the research but not yet described in published literature, software must be made available to editors/reviewers upon request. We strongly encourage code deposition in a community repository (e.g. GitHub). See the Nature Research guidelines for submitting code & software for further information.

Data

Policy information about availability of data

All manuscripts must include a data availability statement. This statement should provide the following information, where applicable:

- Accession codes, unique identifiers, or web links for publicly available datasets
- A list of figures that have associated raw data
- A description of any restrictions on data availability

Data availability statement will be added in the revised manuscript.

Field-specific reporting

Please select the best fit for your research. If you are not sure, read the appropriate sections before making your selection.

☒ Life sciences ☐ Behavioural & social sciences ☐ Ecological, evolutionary & environmental sciences

For a reference copy of the document with all sections, see nature.com/authors/policies/ReportingSummary-flat.pdf

Life sciences study design

All studies must disclose on these points even when the disclosure is negative.

Sample size	Generally, sample sizes were calculated on the assumption that a 30% difference in the parameters measured would be considered biologically significant with an estimate of sigma of 10-20% of the expected mean. Alpha and Beta were set to the standard values of .05 and 0.8, respectively.
Data exclusions	No exclusions.
Replication	All experiments were individually repeated 2-3 times in order to confirm reproducibility.
Randomization	Where applicable animals were randomized to treatment versus control groups. For example, randomized WT mice were performed with Sham or ovariectomy surgery and/or treated with PBS or rAAV9s. WT and SHN3-KO mice were housed all together after weaned from same parents.
Blinding	MicroCT, histology and dynamic histomorphometry were performed by core facilities or individual investigators (Yeon-Suk Yang, Jihea Kim, Jung Min Kim) who were blinded to the nature of the mice under analysis (both what specific mouse strains or treatment groups were in the experiment and whether any individual mouse belonged to control versus experimental groups).

Reporting for specific materials, systems and methods

Materials & experimental systems

n/a	Involved in the study	
☐	☒	Unique biological materials
☐	☒	Antibodies
☐	☒	Eukaryotic cell lines
☒	☐	Palaeontology
☐	☒	Animals and other organisms
☒	☐	Human research participants

Methods

n/a	Involved in the study	
☒	☐	ChIP-seq
☐	☒	Flow cytometry
☒	☐	MRI-based neuroimaging

Unique biological materials

Policy information about availability of materials

Obtaining unique materials	rAAV vectors will be available by purchasing from the Horae Gene Therapy Center at UMass Medical School.

Antibodies

Antibodies used	For Western, the following primary antibodies were used: GFP (1:1000; Takara, 632381), HSP90 (1:1000; Biolegend, 675402), Cre-recombinase (1:1000; Abcam, ab24607).<br><br>For immunofluorescence, anti-Bglap antibody (1:150; Santa Cruz, sc-365797) was used.
Validation	For validation, the following methods were used: 1) use of isotype controls, 2) manufacturer provided validation on the same species.

Eukaryotic cell lines

Policy information about cell lines

Cell line source(s)	ATDC5 cell line was purchased from Sigma.
Authentication	n/a
Mycoplasma contamination	All of our cell lines were tested for mycoplasma contamination in a regular basis (4 times/year) and they were all confirmed to be negative.
Commonly misidentified lines (See ICLAC register)	n/a

Animals and other organisms

Policy information about studies involving animals; ARRIVE guidelines recommended for reporting animal research

Laboratory animals	Shn3-/- mice and Shn3-fl/fl mice were generated as previously described (Jones et al., Science, 2006; Jones et al., PNAS, 2010) and maintained on BALB/cJ and C57BL/6J background, respectively. Osteocalcin-ERT/Cre mice with tamoxifen-induced Cre recombinase expression in mature osteoblasts were generated as previously described (Park et al., Cell Stem Cell, 2012) and crossed with Shn3-fl/fl mice to obtain Shn3-fl/fl ;Ocn-ERT/Cre mice. To label Cre-expressing cells, Shn3-fl/fl ;Ocn-ERT/Cre mice were further crossed with RosamT/mG cre reporter mice (Purchased from Jackson Laboratory). For postnatal activation of ERT/Cre, 100 mg/kg tamoxifen (Sigma) in corn oil (Sigma) was intraperitoneally injected to 2-month-old female mice once a day for 5 consecutive days.
Wild animals	n/a
Field-collected samples	n/a

Flow Cytometry

Plots

Confirm that:

☒ The axis labels state the marker and fluorochrome used (e.g. CD4-FITC).

☒ The axis scales are clearly visible. Include numbers along axes only for bottom left plot of group (a 'group' is an analysis of identical markers).

☒ All plots are contour plots with outliers or pseudocolor plots.

☒ A numerical value for number of cells or percentage (with statistics) is provided.

Methodology

Sample preparation	Femurs and tibias dissected from rAAV9-treated mice were crushed in Hanks Balanced Salt Solution (Life Technologies) containing 10 mM HEPES (pH 7.2) (CellGro) and enzymatically digested with 2.5 mg/mL Collagenase A (Roche) and 1 unit/mL Dispase II (Roche) for 15 min at 37°C under gentle agitation. The resulting cell suspensions were filtered (40 µm), washed using PBS (pH 7.2) containing 0.5% BSA (Fraction V) and 2 mM EDTA. After washing, cells were resuspended in PBS (pH 7.2) with 2 mM EDTA and 1µg/mL 4-6,Diamidino-2-Phenylindole (DAPI) (live/dead exclusion) and EGFP-expressing cells were sorted using a FACS Aria II SORP cell sorter (Becton Dickinson) at the University of Massachusetts Medical School with exclusion of DAPI+ cells and doublets.
Instrument	FACS Aria IISORP cell sorter (Becton Dickinson) for cell sorting.
Software	FlowJo software (TreeStar)
Cell population abundance	It was not feasible to run a purity check on the post-sort population of cells.
Gating strategy	Not included because of a single EGFP sorting.

☐ Tick this box to confirm that a figure exemplifying the gating strategy is provided in the Supplementary Information.
